# Supplementary material for: COVID-19 in the context of pregnancy, infancy and parenting (CoCoPIP) study: protocol for a longitudinal study of parental mental health, social interactions, physical growth and cognitive development of infants during the pandemic
Source: BMJ Open. 2022 Jun 6;12(6):e053800. doi: 10.1136/bmjopen-2021-053800 (PMC9170803; doi:10.1136/bmjopen-2021-053800)
Supplement: Supplementary data [file bmjopen-2021-053800supp001.pdf]

Suppl file 1: Full Questionnaire

CoCoPIP supplementary information

COVID-19 items **2**
Situational influences *2*
Health report *6*
COVID Impact *13*
COVID Concern *13*
Social Distance Impacts *14*
Vaccines *18*
Parental Information **20**
Caregiving support *20*
Pregnancy related anxiety questionnaire – COVID adapted. *21*
Parent infant attachment *22*
Pregnancy information **23**
Healthcare support and access *23*
Parenting, family and home environment **27**
Face-to-face interaction index. *27*
Household space and environment *31*
Family demographics and socioeconomic status *33*

Aydin E, *et al. BMJ Open* 2022; 12:e053800. doi: 10.1136/bmjopen-2021-053800

Suppl file 1: Full Questionnaire

COVID-19 items

Situational influences

We understand that guidance around COVID-19 has been changing, information is/was not always available and variability in health, work and family matters influence individual responses. We will ask you to share with us how local COVID-19 guidance impacted your lifestyle and behaviour.

Think back over the last few months: when you first changed your behaviour due to COVID-19 (start), during the time when there were the most rules and restrictions in your area related to containing COVID-19 (peak), and the gradual relaxing of those rules (reopening).

First, help us understand the timing of when COVID-19 **affected your life.** No need to look it up or be precise, just use your memory to tell us about **what date you pinpoint the following events (dd/mm/yyyy):**

a. Start of COVID-19
b. Peak of COVID-19
c. First day of Reopening

Currently according to the UK government guidance, what local restriction tier has your area been placed in?

a. Tier 1
b. Tier 2
c. Tier 3
d. Tier 4
e. Tier 5
f. I do not live in the UK
g. Not applicable

Suppl file 1: Full Questionnaire

How do the following statements reflect your response and behaviour with respect to changing local COVID-19 guidance?

+Essential goods: food, hygiene, cleaning supplies and medicine

*Social distance: interact with others **further** than public health advice [1-2 m or 6 feet]

Social contact: interact with others **closer than public health advice

Not true at all (1)	True at the Start of COVID-19 (2)	True at the peak of COVID-19 (3)	True during reopening (4)	True during the last month (5)

a. Stayed home or on my property most of the time
b. Stayed home except for obtaining essentials goods+
c. Stayed home except for exercising and essential goods
d. Go on outings alone or with household members, but not seeking social contact with others
e. Social distance* contact with family or friends outside my immediate household
f. Social contact** with limited families or friends outside household
g. Not able to adhere to social distancing in public
h. Social contact** was necessary for my work

Aydin E, *et al. BMJ Open* 2022; 12:e053800. doi: 10.1136/bmjopen-2021-053800

Suppl file 1: Full Questionnaire

Think about when you first heard about COVID-19. What did your local government call the laws or ordinances guiding the changes in conduct, business closure and distance during social interactions?

a. Lockdown
b. Stay-at-home
c. Other

When did you modify your routine to adhere to lockdown

a. I have largely kept my routine the same as before
b. At least a week before the government formally issued it
c. A few days before the government issued it
d. The day the government issued it
e. A few days after the government issued it
f. At least a week after the government issued it

Many regions have lifted or relaxed some of these restrictions. When did you modify your routine to return to normal?

d. At least a week before the government eased restrictions
e. A few days before the government eased restrictions
f. The day the government eased restrictions
g. A few days after the government eased restrictions
h. At least a week after the government eased restrictions
i. I have largely kept my routine the same as before
j. Not applicable

Suppl file 1: Full Questionnaire

How hard was the initial adjustment to a new routine for you, your partner and family during lockdown?

No adjustment (1)	Not hard, Easy or minor (2)	Hard yet Manageable (3)	Quite Hard, Challenging (4)	Unbearable, Devastating (5)

Working remotely
Being responsible for homeschooling
Interfacing with online education or technology for myself, work or my child
Adjusting to lack of or limited childcare
Enforcing new routines and schedules for the family
Missing interactions with loved ones, colleagues and friends in person

How would you describe your current living situation regarding COVID-19?

a. Staying home at all times
b. Staying home except for getting food / medication
c. Staying home except for exercising and getting food / medication
d. Going out of the house for work or for other reasons, but avoiding social contact with others
e. Enjoying social contact with others while adhering to social distancing
f. Not engaging in social distancing

Suppl file 1: Full Questionnaire

Health report

Have you been diagnosed with COVID-19? Select the statement best reflects your experience.

a. No symptoms or reason to suspect I had / have it
b. Asymptomatic, but positive antibody or COVID-19 test
c. May have been exposed from contact with someone who had it, but no symptoms developed
d. Mild, cold-like symptoms or loss of smell, did not much impact daily function
e. Mild to moderate, flu-like symptoms, impaired daily function
f. Moderate, requiring bedrest, impacted daily function
g. Severe, requiring hospital treatment, but not life threatening
h. Life-threatening/critical with no prospect of long-term impact/disability
i. Life-threatening/critical with potential long-term impact/disability

Have you been diagnosed with COVID-19? Select the statement best reflects your experience.

j. No symptoms or reason to suspect I had / have it
k. Asymptomatic, but positive antibody or COVID-19 test
l. May have been exposed from contact with someone who had it, but no symptoms developed
m. Mild, cold-like symptoms or loss of smell, did not much impact daily function
n. Mild to moderate, flu-like symptoms, impaired daily function
o. Moderate, requiring bedrest, impacted daily function
p. Severe, requiring hospital treatment, but not life threatening
q. Life-threatening/critical with no prospect of long-term impact/disability
r. Life-threatening/critical with potential long-term impact/disability

Aydin E, *et al. BMJ Open* 2022; 12:e053800. doi: 10.1136/bmjopen-2021-053800

Suppl file 1: Full Questionnaire

Do you think someone in your household has had, possibly had or currently has COVID-19 (with or without having test results)?

a. Partner
b. Child
c. Other household member
d. No members of my household

How severe were/are their symptoms/disease? Please select only one.

a. No symptoms
b. Mild, cold-like symptoms, did not much impact daily function
c. Mild to moderate, flu-like symptoms, impaired daily function
d. Moderate, requiring bedrest, impacted daily function
e. Severe, requiring hospital treatment, but not life threatening
f. Life-threatening/critical with no prospect of long-term impact/disability
g. Life-threatening/critical with potential long-term impact/disability
h. They lost their life

Suppl file 1: Full Questionnaire

Select the statement that best reflects the experience of your lockdown with COVID-19 symptoms.

a. No symptoms, but positive antibody or COVID-19 test
b. May have been exposed from contact with someone who had it, but no symptoms developed
c. Mild, cold-like symptoms or loss of smell, did not much impact daily function
d. Mild to moderate, flu-like symptoms, impaired daily function
e. Moderate, requiring bedrest, impacted daily function
f. Severe, requiring hospital treatment, but not life threatening
g. Life-threatening/critical with no prospect of long-term impact/disability
h. Life-threatening/critical with potential long-term impact/disability
i. They lost their life

Select the statement that best reflects the experience of your lockdown

a. No symptoms, but positive antibody or COVID-19 test
b. May have been exposed from contact with someone who had it, but no symptoms developed
c. Mild, cold-like symptoms or loss of smell, did not much impact daily function
d. Mild to moderate, flu-like symptoms, impaired daily function
e. Moderate, requiring bedrest, impacted daily function
f. Severe, requiring hospital treatment, but not life threatening
g. Life-threatening/critical with no prospect of long-term impact/disability
h. Life-threatening/critical with potential long-term impact/disability
i. They lost their life

Aydin E, *et al. BMJ Open* 2022; 12:e053800. doi: 10.1136/bmjopen-2021-053800

Suppl file 1: Full Questionnaire

Do you think any one else significant to you *outside your household* has had or currently has COVID-19 or related symptoms (with or without having test results)? Please specify who and their relation to you:

a. No one I am close with
b. Parent
c. Child
d. Close relative (specify)
e. Close friend or community member (specify)
f. Other loved one (specify)

How were they diagnosed or affected by COVID-19? Select the statement that best reflects their experience.

a. No symptoms, but positive antibody or COVID-19 test
b. May have been exposed from contact with someone who had it, but no symptoms developed
c. Mild, cold-like symptoms or loss of smell, did not much impact daily function
d. Mild to moderate, flu-like symptoms, impaired daily function
e. Moderate, requiring bedrest, impacted daily function
f. Severe, requiring hospital treatment, but not life threatening
g. Life-threatening/critical with no prospect of long-term impact/disability
h. Life-threatening/critical with potential long-term impact/disability
i. They lost their life
j. Other relevant information (key worker, in nursing home, health care provider, etc.)

Aydin E, *et al. BMJ Open* 2022; 12:e053800. doi: 10.1136/bmjopen-2021-053800

How has this affected you emotionally? Please rate how hard you found it / are finding it to cope with them having COVID-19:

a. Unbearable, not coping well
b. Very hard, challenging and distressed
c. Hard, but maintaining perspective
d. Not hard, but not an easy adjustment
e. Not hard at all

How well supported do you feel by your spouse/partner during the COVID-19 lockdown?

a. Extremely
b. Very much
c. Somewhat
d. Not very
e. I do not feel supported at all

How well supported do you feel by your friends and family during the COVID-19 lockdown?

a. Extremely
b. Very much
c. Somewhat
d. Not very
e. I do not feel supported at all

Suppl file 1: Full Questionnaire

How difficult have you found it to be separated from these loved ones because of COVID-19?

a. Unbearable
b. Very difficult
c. Moderately difficult
d. Slightly difficult
e. Not affecting you at all

The next series of questions will be related to whether you have had to self-isolate and your experience during your period of self-isolation. Have you self-isolated in response to potential COVID-19 exposure?

a. Yes
b. No

Did you self-isolate with a partner or other family members?

a. Yes (please specify)
b. No

How many days did you self-isolate for?
(numeric response)

Were you able to self-isolate in your own home?

a. Yes
b. No (please specify)

Suppl file 1: Full Questionnaire

How supported did you feel during self-isolation? (please let us know why)

a. not supported at all
b. a little supported
c. somewhat supported
d. very supported

How did you manage caregiving during self-isolation? (e.g., caring for your family) (open ended)

Do you use the NHS track and trace app?

a. Yes
b. No

Approximately how many times have you used this app to 'check-in' to a venue?

Number of times use

0 10 20 30 40 50 60 70 80 90 100

In the last week ()	
In the last month ()	

Aydin E, *et al. BMJ Open* 2022; 12:e053800. doi: 10.1136/bmjopen-2021-053800

Suppl file 1: Full Questionnaire

COVID Impact

Relative to others, rate how hard you find it to cope with the impact of COVID-19 on the following aspects of your life:

Not hard at all, coping well (1)	Not hard, but not an easy adjustment (2)	Hard, but maintaining perspective (3)	Very hard, challenging & distressed (4)	Unbearable, not coping well (5)

Physical health

Psychological well-being

Social life (or lack thereof)

Financial uncertainty

Disruption to routine

Adjustments to household

Changes in employment

COVID Concern

When answering the next set of questions, please answer them in relation to your feelings since your government implementd a 'stay-at-home' or lockdown request because of COVID-19.

Not at all (1)	A little (2)	Somewhat (3)	Quite a bit (4)	Very much (5)	All the time (6)

I think about the effect of COVID-19 on my own physical health.

I worry about spreading COVID-19 to others.

I am concerned about the effects of lockdown on society.

Lockdown affects/affected my mental health.

I am afraid of COVID-19.

I am not worried about COVID-19.

I am worried that I or people I love will get sick from COVID-19.

I am stressed around other people because I worry I'll catch COVID-19.

I have tried hard to avoid other people because I don't want to get sick

I have tried hard to avoid other people because I don't want to make others sick.

Suppl file 1: Full Questionnaire

Social Distance Impacts

How much do each of the following statements reflect your attitude or experience during COVID-19?

Not at all (1)	A little (2)	Somewhat (3)	Quite a bit (4)	Very true (5)

I spend more time alone

There is a sense of solidarity in my community

I am even busier than before.

I have volunteered to get groceries for neighbors or acquaintances

I feel grateful for my health

I have more time than usual

There is more fighting among the members of my household

I have picked up a new hobby

I am learning a new skill

I appreciate moments of reprieve from the chaos in my home and outside it.

Technology brings us together during these turbulent

I spend my leisure time more intentionally times.

I am exercising or appreciating outdoor time more often.

I feel proud of my community's response

I am hopeful that we can learn something out of this.

I am disappointed by the changes in observing special occasions and holidays

I am concerned for the health of an elderly or vulnerable family or friend due to COVID-19. I am concerned for the well-being of a family or friend due to social distancing.

I am now more conscious about my personal space boundaries when outside my home.

I am just as cautious and vigilant in maintaining distance from others in public as I was at the start of the outbreak

Suppl file 1: Full Questionnaire

Do you think someone in your household has had, possibly had or currently has COVID-19 (with or without having test results)?

e. Partner
f. Child
g. Other household member
h. No members of my household

How severe were/are their symptoms/disease? Please select only one.

i. No symptoms
j. Mild, cold-like symptoms, did not much impact daily function
k. Mild to moderate, flu-like symptoms, impaired daily function
l. Moderate, requiring bedrest, impacted daily function
m. Severe, requiring hospital treatment, but not life threatening
n. Life-threatening/critical with no prospect of long-term impact/disability
o. Life-threatening/critical with potential long-term impact/disability
p. They lost their life

Aydin E, *et al. BMJ Open* 2022; 12:e053800. doi: 10.1136/bmjopen-2021-053800

Select the statement that best reflects the experience of your lockdown with COVID-19 symptoms.

j. No symptoms, but positive antibody or COVID-19 test
k. May have been exposed from contact with someone who had it, but no symptoms developed
l. Mild, cold-like symptoms or loss of smell, did not much impact daily function
m. Mild to moderate, flu-like symptoms, impaired daily function
n. Moderate, requiring bedrest, impacted daily function
o. Severe, requiring hospital treatment, but not life threatening
p. Life-threatening/critical with no prospect of long-term impact/disability
q. Life-threatening/critical with potential long-term impact/disability
r. They lost their life

Select the statement that best reflects the experience of your lockdown

j. No symptoms, but positive antibody or COVID-19 test
k. May have been exposed from contact with someone who had it, but no symptoms developed
l. Mild, cold-like symptoms or loss of smell, did not much impact daily function
m. Mild to moderate, flu-like symptoms, impaired daily function
n. Moderate, requiring bedrest, impacted daily function
o. Severe, requiring hospital treatment, but not life threatening
p. Life-threatening/critical with no prospect of long-term impact/disability
q. Life-threatening/critical with potential long-term impact/disability
r. They lost their life

Do you think any one else significant to you *outside your household* has had or currently has COVID-19 or related symptoms (with or without having test results)? Please specify who and their relation to you:

g. No one I am close with
h. Parent
i. Child
j. Close relative (specify)
k. Close friend or community member (specify)
l. Other loved one (specify)

How were they diagnosed or affected by COVID-19? Select the statement that best reflects their experience.

k. No symptoms, but positive antibody or COVID-19 test
l. May have been exposed from contact with someone who had it, but no symptoms developed
m. Mild, cold-like symptoms or loss of smell, did not much impact daily function
n. Mild to moderate, flu-like symptoms, impaired daily function
o. Moderate, requiring bedrest, impacted daily function
p. Severe, requiring hospital treatment, but not life threatening
q. Life-threatening/critical with no prospect of long-term impact/disability
r. Life-threatening/critical with potential long-term impact/disability
s. They lost their life
t. Other relevant information (key worker, in nursing home, health care provider, etc.)

How has this affected you emotionally? Please rate how hard you found it / are finding it to cope with them having COVID-19:

f. Unbearable, not coping well
g. Very hard, challenging and distressed
h. Hard, but maintaining perspective
i. Not hard, but not an easy adjustment
j. Not hard at all

Vaccines

Are you currently eligible for the COVID-19 vaccine?

a. Yes
b. No
c. Unsure
d. Prefer not to answer
e. Not applicable

Have you and/or your partner been vaccinated?

a. Yes
b. No
c. Not applicable

Have any of your family members or loved ones been vaccinated?

a. Yes (please specify who)
b. No
c. Unsure

Do you think that getting vaccinated has changed the way you socialise?

a. Yes
b. No
c. Prefer not to answer

Could you tell us how and/or why getting vaccinated has changed the way you socialise? (open ended)

Suppl file 1: Full Questionnaire

Parental Information

Caregiving support

Please tell us who has served as caregivers to your baby since their birth. Please select all that apply.

Every day (1)	A few times a week (2)	Several times a month (3)	Once a month (4)	Never or less than once a month (5)

a. My partner/spouse
b. My parents or in-laws
c. My grandparents or grandparents-in-law
d. Other relatives (siblings, aunts/uncles, cousins etc.)
e. Close friends
f. Nanny/Babysitter
g. Other (please specify)

During an average night, how many hours does your baby sleep? (scale 0-8 hours)

a. Before COVID-19
b. Since COVID-19

If your baby sleeps more than 8 hours, please specify below:
(open ended)

On an average day during the week, how many hours would you say you spend with your baby between 7am and 7pm? (scale 0-12 hours)

a. Before COVID-19
b. Since COVID-19

In an average month, how many people other than caregivers and household members have interacted with your baby? (scale 0-30)

a. Before COVID-19 (number of people
b. Since COVID-19 (number of people)

Suppl file 1: Full Questionnaire

When you and your baby are together, how often do you:

A lot (1)	Often (2)	Sometimes (3)	Rarely (4)	Never (5)

a. Hold your baby
b. Sing to your baby
c. Soothe your baby
d. Talk to your baby
e. Read to your baby
f. Imitate your baby
g. Bathe your baby
h. Stroke your baby
i. Cuddle your baby
j. Kiss your baby
k. Play with your baby (with toys)
l. Play face to face with your baby (without toys)
m. Play with your baby (with screens, e.g. iPad)
n. Play with your baby (using household objects)
o. Watch your baby play
p. Watch your baby sleep

a. Take your baby outside
b. Take your baby for a walk in a pram/stroller

Pregnancy related anxiety questionnaire – COVID adapted.

We are going to ask how much you think or worry about **your pregnancy or the experience of your pregnant partner during birth.** Please slide the bar that most closely matches your feelings about each statement.

Not at all (1)	A little (2)	Somewhat (3)	Quite a bit (4)	Very much (5)

a. I worry about the pain of contractions
b. I worry about the pain of delivery
c. I worry about not being in control of my body while I give birth, or how my partner will respond while giving birth (shouting, crying, bowel movement, etc.)
d. I think about coming in contact with COVID-19

Aydin E, *et al. BMJ Open* 2022; 12:e053800. doi: 10.1136/bmjopen-2021-053800

e. I think about the pain from getting COVID-19
f. I think about difficulty accessing health care if there is a virus spreading
g. I think that something will be wrong with my baby physically
h. I think that something will be wrong with my baby mentally
i. I think I will transmit stress about COVID-19 to my baby
j. I think due to COVID-19 my baby may be held back
k. I think due to COVID-19 my baby may not be normal
l. I think due to COVID-19 my baby may not get proper medical attention
m. I think due to COVID-19 my birth will have complications
n. I think that after birth my child will be in poor health
o. I think about creating a virus-free home for my child
p. I think about creating a safe, healthy home for my child
q. Thinking about COVID-19 makes me feel threatened
r. I think my baby will not be healthy due to COVID-19
s. I think I can transmit COVID-19 to my baby
t. COVID-19 has made me rethink my family planning

Home schooling

'Could you describe your experience of home schooling during your pregnancy or as a new parent?'

(open ended)

Parent infant attachment

Could you tell us a little about what you imagine your baby will be like and how the two of you will get along?

(open ended)

Could you tell us a little about what your baby and how the two of you get along?

(open ended)

Suppl file 1: Full Questionnaire

Pregnancy information

Healthcare support and access

Have you attended some or all midwife, doctor or OB-GYN pregnancy appointments in person?

a. Yes
b. No

Do you feel comfortable attending your pregnancy appointments?

a. Yes
b. A little
c. Unsure
d. Not at all

Could you please tell us why you chose the answer you did for the last question?
(open ended)

Have you been offered online, phone or video call midwife appointments?

a. Yes
b. No

Do you feel that talking to your midwife online has allowed you to ask the questions you've wanted to and made you feel at ease?

a. Yes
b. A little
c. Unsure
d. Not at all

Could you tell us why you chose the answer you did for the last question?
(open ended)

Suppl file 1: Full Questionnaire

How well supported do you feel by your midwife, doctor (OB-GYN) and other prenatal healthcare professionals during this time?

a. Extremely
b. Very much
c. somewhat
d. not very
e. I do not feel supported at all

Could you tell us about the support from your healthcare providers during your pregnancy?
(open ended)

When attending your pregnancy appointments, have you worn a mask, gown or other form of personal protective equipment (PPE)? Check all that apply.

a. No, I nor my healthcare providers used PPE
b. No I did not, though the healthcare staff did wear PPE
c. The healthcare staff provided me with PPE (please specify, gloves, gown, etc.)
d. I brought a face covering or mask with me to the appointment
e. I brought additional PPE with me to the appointment (please specify, gloves, gown, etc.)

How comfortable did you feel interacting with your healthcare providers at these appointments?
(open ended)

Suppl file 1: Full Questionnaire

Have you had any appointments cancelled or rescheduled?

a. No
b. Rescheduled by health provider
c. Cancelled by health provider
d. I did not attend due to safety or health concerns
e. I cancelled due to other reasons
f. I have not booked any appointments since COVID-19 due to safety or health concerns

Are you attending antenatal classes? (Either online or in person)

a. Yes (in person)
b. Yes (online)
c. No

Are the classes you are taking offered by public, private and alternative healthcare providers? Please tick and specify what kind of classes you took.

a. Private antenatal class (e.g. NCT classes, small business, offered by university or employer, etc.)
b. Not private, provided by or subsidized by the public healthcare system (e.g. NHS, Medicare)
c. Mental health services (counselling, family planning, genetic counselling, etc.)
d. Other, including alternative wellness services (yoga, doula, etc.)

Aydin E, *et al. BMJ Open* 2022; 12:e053800. doi: 10.1136/bmjopen-2021-053800

In your own words, please tell us about these classes; how often you attend and how helpful you have found them.
(open ended)

Are these classes supporting you in the same way (if you had a previous pregnancy) or in the way you expected during your pregnancy?

a. Yes
b. No (please specify)
c. I am not sure

Suppl file 1: Full Questionnaire

Parenting, family and home environment

Face-to-face interaction index.

Think about who your baby has interacted with so far, and who would have interacted with your baby in person if not for COVID-19. We want to get an understanding of your baby's visual and physical social interactions. Who would you have expected to see your baby (visit, talk, make silly faces, play from afar) or hold your baby (e.g. rock, play with your baby)? Please select all that apply.

Actually interacted with my baby (1)			If not for COVID-19, would have interacted with my baby (2)		
See from afar (1.1)	Hold up close (1.2)	Via videochat (1.3)	See from afar (2.1)	Hold up close (2.2)	Via videochat (2.3)

a. My partner
b. My parents
c. My in-laws
d. My or my partner's grandparents
e. Other adult relatives
f. Friends
g. Colleagues
h. Nanny/Babysitter
i. Neighbors
j. Healthcare or prenatal service provider
k. Other children

Think about how often you and your baby have interacted with friends and family, both in person and on video in 2020. Think about the time **before COVID-19, right after the start of COVID-19 in your region, and in the last month.**

Aydin E, *et al. BMJ Open* 2022; 12:e053800. doi: 10.1136/bmjopen-2021-053800

Suppl file 1: Full Questionnaire

How often did you (without your baby) videochat with others (for work, family or social reasons)?

Never (1)	A handful of times (2)	At least once a month (3)	At least once a week (4)	At least once day (5)	Multiple times a day (6)	Not Applicable (7)

a. Before COVID-19
b. Start of COVID-19
c. Last month

In a typical month, how often did you record videos of your baby using your smartphone?

Never (1)	A handful of times (2)	At least once a month (3)	At least once a week (4)	At least once day (5)	Multiple times a day (6)	Not Applicable (7)

a. Before COVID-19
b. Start of COVID-19
c. Last month

How often did you AND your baby interact in person with friends or family, other than caregivers?

Never (1)	A handful of times (2)	At least once a month (3)	At least once a week (4)	At least once day (5)	Multiple times a day (6)	Not Applicable (7)

a. Before COVID-19
b. Start of COVID-19
c. Last month

Aydin E, *et al. BMJ Open* 2022; 12:e053800. doi: 10.1136/bmjopen-2021-053800

Suppl file 1: Full Questionnaire

Using your phone, do you share videos or pictures of your baby with (select all that apply):

a. your sibling(s)
b. your parents (baby's grandparents)
c. other relatives
d. friends
e. colleagues
f. private online network (e.g., baby website)
g. public online network (e.g., personal blog)
h. private social media (e.g., instagram, facebook)
i. public social media (e.g., instagram, facebook)
j. None of the above

How responsive is your baby to others during interactions on videochat, Zoom, facetime or webcam?

a. Very responsive
b. Quite responsive
c. Somewhat
d. Rarely
e. Never responds
f. Not applicable

Suppl file 1: Full Questionnaire

How responsive is your baby to videos, music or other media played from your mobile device?

a. Most of the time
b. Quite a bit
c. Somewhat
d. Rarely
e. Never responds
f. Not applicable

Have you captured videos of your baby's milestones or behaviours? Please select all that apply

a. No
b. Reaching
c. Grasping
d. Sitting up
e. Rolling over
f. Smiling
g. Babbling
h. Waving
i. Other

Suppl file 1: Full Questionnaire

Household space and environment

Have you been living and/or staying in the same home since Feb 2020? Since this time have you:

a. Moved home
b. Visited friends or family
c. Gone to stay with family
d. Gone on holiday
e. Other (please specify)
f. None of the above

How many people lived with you in your current residence?

Before Covid-19 (1)	Peak Covid-19 (2)	Presently (3)

Number of Adults

Number of Children (under the age of 18)

Number of adults with conditions which confer risk for or complications with COVID-19

How many rooms within your current residence do you feel comfortable relaxing or spending time in that are not your bedroom? This can also include outdoor spaces that are on your property.

a. < 2
b. 2-3
c. 3-5
d. 5-8
e. 8+

Aydin E, *et al. BMJ Open* 2022; 12:e053800. doi: 10.1136/bmjopen-2021-053800

Do you currently have access to outside space you feel safe spending time in? Please select all that apply.

a. Balcony or backyard
b. Communal garden or yard
c. Small private garden or yard
d. Large private garden or yard
e. Allotment
f. Parks
g. Playgrounds
h. Nearby countryside or field
i. National park, beach or other outdoor space
j. Other (please specify)

In your home, how many televisions are there in total?

a. < 2
b. 2-3
c. 3-5
d. 5-8
e. 8+

In your home, how many mobile phones are presently in use?

a. < 2
b. 2-3
c. 3-5
d. 5-8
e. 8+

Suppl file 1: Full Questionnaire

How many personal computers and/or tablets are presently in use?

a. < 2
b. 2-3
c. 3-5
d. 5-8
e. 8+

Family demographics and socioeconomic status

Are you or your partner currently pregnant?

a. Yes
b. No

Have you had your first trimester scan?

a. Yes
b. No

Are you the parents to an infant under the age of 7 months?

a. Yes
b. No

How old is your child?

a. Months
b. Days

Who is completing this questionnaire?

a. Mother
b. Father
c. Non birth mother
d. Other (please specify)

Suppl file 1: Full Questionnaire

What is your ethnic background? You may select more than one.

a. White
b. Black
c. Asian
d. Mixed/multiple ethnic group (e.g. white and African)
e. Hispanic
f. Arab
g. Other (please specify)
h. Prefer not to answer

How old are you?

(numeric response)

What is the mother's highest level of education?

a. High School/ Secondary School (or something equivalent) to age 16
b. College level education (to age 18)
c. Undergraduate degree
d. Postgraduate degree (Masters, PhD)

What is the father's/partner's highest level of education?

a. High School/ Secondary School (or something equivalent) to age 16
b. College level education (to age 18)
c. Undergraduate degree
d. Postgraduate degree (Masters, PhD)

What is your post code?

(open ended optional)

Aydin E, *et al. BMJ Open* 2022; 12:e053800. doi: 10.1136/bmjopen-2021-053800

Suppl file 1: Full Questionnaire

What is your current employment status? Please select ALL that apply

a. On maternity leave
b. Unemployed (or job seeking)
c. Unemployed due to COVID-19
d. Full-time home-maker
e. Retired
f. Employed full-time (working from home due to COVID-19)
g. Employed full-time (I am a key worker)
h. Employed full time (with reduced hours and salary loss)
i. Employed full-time (with reduced hours but without salary loss)
j. Employed with reduced hours due to childcare/homeschooling needs as a result of COVID-19
k. Employed (part-time) with reduced hours and salary loss
l. Employed (part-time) with reduced hours but without salary loss
m. Furloughed (with salary) - Full-time
n. Furloughed (without salary) - Full-time
o. Furloughed (with salary) - Part-time
p. Furloughed (without salary) - Part-time
q. Self-employed (currently still working)
r. Self-employed (unable to work due to COVID-19)
s. Self-employed with reduced hours and salary loss
t. Self-employed with reduced hours but without salary loss
u. Student - My studies have not been affected by the COVID-19
v. Student - I have had to postpone my studies due to COVID-19
w. Student - My studies have been negatively impacted by the COVID-19
x. Other (please specify)

Aydin E, *et al. BMJ Open* 2022; 12:e053800. doi: 10.1136/bmjopen-2021-053800
