# Supplementary material for: COVID-19 in the context of pregnancy, infancy and parenting (CoCoPIP) study: protocol for a longitudinal study of parental mental health, social interactions, physical growth and cognitive development of infants during the pandemic
Source: BMJ Open. 2022 Jun 6;12(6):e053800. doi: 10.1136/bmjopen-2021-053800 (PMC9170803; doi:10.1136/bmjopen-2021-053800)
Supplement: Supplementary data [file bmjopen-2021-053800supp002.pdf]

Measure (items)	Description	Scale	Example item
**COVID-19 items**			
Situational influences	Assessing adjustment and adherence to government guidelines.		'When did you modify your routine to adhere to the social distancing guidance?'
Health report	Assessing risk for COVID exposure and symptoms of parents, partner and/or social network. As well as self-isolation behaviours and support (if applicable)		'Have you been diagnosed with COVID?' 'How supported did you feel during self-isolation?'
COVID Impact (7 items)	How COVID-19 has impacted themselves and their household (e.g., physical health, disruption to routine).	Five-point scale from 'not hard at all, copying well' to 'unbearable, not coping well'	
COVID Concern (10 items)		Six-point scale from 'not at all' to 'all the time'	'I am not worried about COVID-19'
Event Impact Scale-Revised (20 items) †	Assessing the distress experience as a result of traumatic events	Five-point scale from 'extremely' to 'not at all'	'I felt irritable and angry'
Social distance impacts	Assessing participants attitudes and experience during COVID-19.	Five-point scale from 'not at all' to 'very true'	'I spend my leisure time more intentionally'
Vaccines	Assessing access to and attitude towards a vaccine and potential changes in social behaviour in relation to a vaccine.		'Are you currently eligible for the COVID-19 vaccine?' / 'Could you tell us how and/or why getting vaccinated has changed the way you socialise?'
**Parental information**			
State-Trait Anxiety state (STAI-S) (20 items) †	Assessing the presence and severity of an individual's state-based anxiety (i.e., anxiety in response to what is happening right now).	Four-point scale from 'not at all' to 'very much so'	'I feel strained'
Caregiving support	Questions assessing remote and in person availability of household members, partners, and social network of families		
Home schooling	Exploring the additional demands on parents in the home and support available for home schooling.		'Could you describe your experience of home schooling during your pregnancy or as a new parent?'
Stress Life Inventory (43 items) †	Assessing the number of stressful life events experienced by the family over a 12-month period	Items rates 'yes' or 'no'	'Being fired at work'

Pregnancy related anxiety questionnaire (PRAQ) (20 items)*	Assessing pregnancy-specific anxiety in women during the antenatal period.	Five-point scale from 'not at all' to 'very much'	'I think that after birth my child will be in poor health' / 'I think about creating a virus-free home for my child'
**Pregnancy information**			
Healthcare support and access	Exploring parental experiences of their healthcare support and care, and access to antenatal routine care during the pandemic		'Do you feel comfortable attending your pregnancy appointments?'/ 'Could you tell us about the support from your healthcare provides during your pregnancy'
Antenatal Emotional Attachment Scale (AEAS) - Maternal (19 items) †	Questions asking expectant parents about their thoughts and feelings over the last two-week period about their baby	Responses on a varying five-point scale (e.g., 'almost all the time' to 'not at all' and 'very positive' to 'very negative')	'I think of the developing baby as mostly as;'
Antenatal Emotional Attachment Scale (AEAS) – Paternal (16 items) †			'I have found myself touching, or rubbing with my hand, the outside of my partners stomach where the baby is:'
**Toddler outcomes**			
Infant Behaviour Questionnaire (IBQ) – short form (36 items) †	Measures the dimensions of temperament in children younger than 12 months of age	Eight-point scale from 'never' to 'always' (including option for 'does not apply')	'When tired, how often did your baby show distress?'
Infant/Toddler Sensory Profile (ITSP) (36 items) †	A caregiver report on how responsive, sensitive, avoidant and alert their babies are when presented with stimuli visual, auditory, tactile and vestibular modalities	Six-point scale from 'almost always' to 'almost never' (including an option for 'does not apply')	'My child is active throughout the day'
Ages and Stages (ASQ-3) (39 items) †	A developmental screening tool assessing a child's developmental progress in six domains (i.e., communication, gross motor, fine motor, problem solving and personal-social development).	Three-point scale from 'yes' to 'not yet'	'When your baby wants something, does he tell you by pointing to it?'
Oxford Communication Development Inventory – 100 words (CDI) †	A UK-adapted measure of toddler language development	Two-point scale; 'understands' and 'understands and says'	'car'
Quantitative Checklist for Autism in Toddlers (Q-CHAT) (25 items) †	A quantitative measure of early autistic traits in toddlers	Responses given on a varying five-point scale (e.g., 'many times a day to never' and 'always' to 'never')	'How easy is it for your child to adapt when his/her routine changes or when things are out of their usual place?'
Vineland parent and caregiver form†	A number of questions about the home environment and family-life behaviour	Three-point scale from "usually or often' to 'never'	'When you look or point at something, looks in that direction.'

**Parenting, family and home environment**			
Household space and environment	Assessing access to outdoor/indoor space and technology		'Do you currently have access to outdoor space you feel safe spending time in?' / 'In your home, how many mobile phones are presently in use?'
Parent-infant attachment	Parental description of relationship with infant (imagined and actual)		'Could you tell us a little about what you image your baby will be like and how the two of you will get along?'
Face-to-face interaction index	Exploring the frequency of face-to-face and distant interaction the infant has had since birth		'How often did you with/without your baby video chat with others?'/ 'How responsive is your baby to others during virtual interactions?'
Parenting reflective functioning questionnaire (PRFQ) (18 item) †	A short measure assessing parental reflective functioning or mentalising.	Responses are rated on a seven-point scale from 'strongly disagree' to 'strongly agree'	'I always know why my child acts the way he or she does'
Comprehensive early childhood parenting questionnaire (CECPAQ) (54 items) †	A parent report measure assessing five domains of parenting (i.e., support, stimulation, structure, harsh discipline and positive measure).	Responses are rated on a six-point scale from 'never' to 'always'	'When my child is having a hard time, I am able to help him/her.
Difficulties in Emotion Regulation Scale (DERS) (19 items) †	A self-report measure of subjective emotion ability	Responses are rated on a five-point scale from 'almost never' to 'almost always'	'When I'm upset, I can still get things done'
Family demographics and socioeconomic questions	Parent and infant age or gestation of pregnancy; sex; ethnicity; location; parent and partner highest level of qualification; employment status and household income.		'What is your highest level of education?'
Postcode	Used to derived indices of multiple deprivation.		

*several questions have been adapted for use during COVID-19

†These items have not been included within the supplementary information as they are standardised questionnaires and not specifically created for this study.

Supp Table 1: A detailed summary of measures used within the study.

BMJ Publishing Group Limited (BMJ) disclaims all liability and responsibility arising from any reliance placed on this supplemental material which has been supplied by the author(s)
