# Supplementary material for: COVID-19 in the context of pregnancy, infancy and parenting (CoCoPIP) study: protocol for a longitudinal study of parental mental health, social interactions, physical growth and cognitive development of infants during the pandemic
Source: BMJ Open. 2022 Jun 6;12(6):e053800. doi: 10.1136/bmjopen-2021-053800 (PMC9170803; doi:10.1136/bmjopen-2021-053800)
Supplement: Author's manuscript [file bmjopen-2021-053800.draft_revisions.pdf]

BMJ Open is committed to open peer review. As part of this commitment we make the peer review history of every article we publish publicly available.

When an article is published we post the peer reviewers' comments and the authors' responses online. We also post the versions of the paper that were used during peer review. These are the versions that the peer review comments apply to.

The versions of the paper that follow are the versions that were submitted during the peer review process. They are not the versions of record or the final published versions. They should not be cited or distributed as the published version of this manuscript.

BMJ Open is an open access journal and the full, final, typeset and author-corrected version of record of the manuscript is available on our site with no access controls, subscription charges or pay-per-view fees (http://bmjopen.bmj.com).

If you have any questions on BMJ Open's open peer review process please email info.bmjopen@bmj.com

BMJ Open

# The COVID in the Context of Pregnancy, Infancy and Parenting (CoCoPIP) Study: protocol for a longitudinal study of parental mental health, social interactions, physical growth, and cognitive development of infants during the pandemic.



| | |
|---|---|
| Journal: | *BMJ Open* |
| Manuscript ID | bmjopen-2021-053800 |
| Article Type: | Protocol |
| Date Submitted by the Author: | 25-May-2021 |



| | |
|---|---|
| Complete List of Authors: | Aydin, Ezra; University of Cambridge, Department of Psychology<br>Weiss, Staci; University of Cambridge, Department of Psychology<br>Glasgow, Kevin; University of Cambridge, Department of Education<br>Barlow, Jane; University of Oxford, Department of Social Policy<br>Austin, Topun; Rosie Hospital; NIHR Cambridge Biomedical Research Centre<br>Johnson, Mark H.; University of Cambridge, Department of Psychology<br>Lloyd-Fox, Sarah; University of Cambridge, Department of Psychology |



| | |
|---|---|
| Keywords: | COVID-19, QUALITATIVE RESEARCH, Fetal medicine < OBSTETRICS |
| | |


SCHOLARONE™
Manuscripts

# The COVID in the Context of Pregnancy, Infancy and Parenting (CoCoPIP) Study: protocol for a longitudinal study of parental mental health, social interactions, physical growth, and cognitive development of infants during the pandemic.

Ezra Aydin, PhD[1*], Staci M. Weiss, PhD[1*], Kevin A. Glasgow, MPhil[2], Jane Barlow, PhD[3], Topun Austin, MD[4,5], Mark H. Johnson, PhD[1] and Sarah Lloyd-Fox, PhD[1]

[1]Department of Psychology, University of Cambridge, UK.

[2]Department of Education, University of Cambridge, UK.

[3]Centre for Evidence-Based Intervention, Department of Social Policy and Intervention, University of Oxford, UK.

[4]The Rosie Hospital, Cambridge University Hospitals Foundation Trust, Cambridge, UK.

[5]NIHR Cambridge Biomedical Research Centre, Cambridge, UK.

*Joint first author.

Correspondence to: Ezra Aydin, Department of Psychology, University of Cambridge, Downing Street, Cambridge, CB2 3EB, UK. Email: ea420@cam.ac.uk Telephone: +44 (0) 1223 465 222

Submission to: BMJ Open

Word limit: 4000

Current word count: 3650

Table count:2

Figure count: 3

For peer review only


For peer review only - http://bmjopen.bmj.com/site/about/guidelines.xhtml

# ABSTRACT

**Introduction** While the secondary impact of the COVID pandemic on the psychological wellbeing of pregnant women and parents has become apparent over the past year, the impact of these changes on early social interactions, physical growth and cognitive development of their infants is unknown, as is the way in which a range of COVID related changes have mediated this impact. This study (CoCoPIP) will investigate: i) how parent's experiences of the social, medical, and financial changes during the pandemic have impacted pre and postnatal parental mental health and parent-infant social interaction; and (ii) the extent to which these COVID-related changes in parental pre and postnatal mental health and social interaction are associated with fetal and infant development.

**Methods and analysis** The CoCoPIP study is a national online survey initiated in July 2020. This ongoing study (n = 1700 families currently enrolled as of 6th May 2021) involves both quantitative and qualitative data being collected across pregnancy and infancy. It is designed to identify the longitudinal impact of the pandemic from pregnancy to two years of age, with the aim of identifying if stress-associated moderators (i.e., loss of income, COVID-19 illness, access to ante/postnatal support) impact parental mental health, and in turn, infant development. In addition, we aim to document individual differences in social and cognitive development in toddlers who were born during restrictions intended to mitigate COVID-19 spread (e.g., social distancing, national lockdowns).

**Ethics and dissemination** Ethical approval was given by the University of Cambridge, Psychology Research Ethics Committee (PREC) (PRE.2020.077). Findings will be made available via community engagement, public forums (e.g., social media,) and to national (e.g., NHS England) and local (Cambridge Universities Hospitals NHS Foundation Trust) healthcare partners. Results will be submitted for publication in peer-reviews journals.




For peer review only - http://bmjopen.bmj.com/site/about/guidelines.xhtml

***Strengths and Limitations of this study***

- This is a new cohort of families being followed from prenatal to postnatal (up to 18 months) during the COVID-19 pandemic.
- The study involves the collection of quantifiable data to identify the short- and long-term influences of the pandemic on key aspects of infant development.
- The study also has a range of open-ended questions for qualitative analysis aimed at exploring familial experiences in more detail.
- The data is being collected online and is therefore limited to self- and parent-report measures, with no direct assessment of child development and parental mental health.
- Although the sample of families being recruited are diverse in their indices of multiple deprivation (IMD) and geographic location, they may not be fully representative of the wider population.

For peer review only

# INTRODUCTION

The COVID-19 pandemic has resulted in an unprecedented challenge to existing medical, social, and economic institutions, akin to prior natural disasters, war or other periods of hardship, with potentially similar long-term implications for the early development and lifelong health of children whose mothers were pregnant or newly delivered during these periods of significant social and economic upheaval [1]. This is due to the fact that the fetal physiological environment and infant caregiving and social environment are now recognised to play a key role in influencing later biological, physical and neurodevelopmental outcomes [2,3]. With regard to the COVID-19 pandemic, the social distancing restrictions and national lockdowns that were put in place to mitigate its transmission, have had a range of secondary consequences impacting the psychological wellbeing of pregnant women and new parents and the postnatal environment that the infant is born into [4–6]. The shifts in socialization, stress and socio-economic position associated with COVID-19 public health guidance may have exacerbated the feelings of vulnerability, health vigilance and isolation associated with the adjustment to parenting. Heightened anxiety and depression were reported during the national lockdown in the United Kingdom [7], with expectant and new mothers experiencing unique physical and psychological stressors [4] as well as constrained access to resources, especially with regard to family and caregiving support. However, little is currently known about the impact of these COVID-related changes on the development of the infant. The current study aims to address this evidence gap by exploring the relationship regarding the family's reported experiences of these changes in terms of their impact on their pre and postnatal mental health, and interaction with their infant, and the potential subsequent impact of these changes on the infant's physical, sensory, affective and cognitive development.

## The secondary impact of COVID-19 on pregnant women

The COVID-19 pandemic has been the biggest public health emergency for over a century, necessitating extreme measures at a societal level to mitigate against death and prevent acute health services from being overwhelmed. However, resulting from this have been a number of secondary consequences ( (i.e., increased caregiving demands for children and family members; isolation from family and community due to social distancing; job loss; financial hardship and increased interpersonal stressors or relationship violence [8]), having a disproportionate and significant impact on women of childbearing age [4]. In the UK, the impact on this group of women has also been exacerbated by National Health Service (NHS) guidance that was produced in response to the national lockdown restrictions [9–11], in which hospital-based midwifery services placed limitations on partners being present during ultrasound visits and birth. In addition, most community-based services were discontinued, other than antenatal contact and new baby visits, all of which were required to be provided virtually unless otherwise indicated, with all other contacts being assessed and stratified according to vulnerability or clinical need (e.g., maternal mental health).



For peer review only - http://bmjopen.bmj.com/site/about/guidelines.xhtml

These changes not only affected the capacity of practitioners to support women during the perinatal period at a time of significantly heightened stress/distress [6] but also resulted in significant regional variations in access to healthcare and advice for expectant mothers across the UK. The changes have been associated with (i) a fourfold increase in stillbirths attributed to lack of preventive antenatal care [12], (ii) birthing partners denied access to the hospital for the birth or asked to leave immediately following the birth, and (iii) limited access to babies admitted to neonatal intensive care. The NHS has also reported a reluctance on the part of parents to attend postnatal GP checks, due to parental attitudes related to COVID-19 infection (Institute of Health Visiting).

**The impact of COVID-19 on parental mental health and parent-infant interaction**

Several online surveys conducted during the first national lockdown indicated that there was a significant increase in antenatal anxiety both in terms of pandemic-related pregnancy stress associated with feeling unprepared for birth due to the pandemic, and stress related to fears of perinatal COVID-19 infection, with one large US survey (n = 4451) showing that around 30% of pregnant women experienced both types of stress [13]. Another US survey (n = 2740) that examined wider sources of stress showed that more than half of women reported increased stress about food running out (59.2%, n = 1622), losing a job or household income (63.7%, n = 1745), or loss of childcare (56.3%, n = 1543). More than a third reported increased stress about conflict between household members (37.5%, n = 1028); and 93% (n = 2556) reported increased stress about getting infected with COVID-19 [14].

A number of online cross-sectional surveys found significantly increased rates of anxiety and depression, based on the use of self-report standardised measures (e.g., Edinburgh Postnatal Depression Scale (EPDS); Hospital Anxiety and Depression Scale (HADS)). For example, a cross-sectional survey of 1,987 pregnant women in Canada in April 2020, found substantially elevated anxiety and depression symptoms, compared to similar pre-pandemic pregnancy cohorts; 37% reported clinically relevant symptoms of depression, and 57% reported clinically relevant symptoms of anxiety [15]. A second Canadian study found that a cohort of pregnant women, who were recruited during the COVID-19 pandemic, were twice as likely to present clinically significant levels of depressive and anxiety symptoms compared with a cohort of pregnant women recruited prior to the pandemic [16]. Early evidence in the UK similarly suggests that the impact on the mental health of pregnant women has been significant with heightened anxiety and depression being reported during the national lockdown (levels of mental distress rising from 18.9% (2018-19) to 27.3% in April 2020, one month into the national lockdown) [4].


For peer review only - http://bmjopen.bmj.com/site/about/guidelines.xhtml

This is of concern because there is consistent evidence to suggest that anxiety and depression in pregnancy can have a long-term impact on child development. For example, recent systematic reviews found that antenatal anxiety is associated with a range of adverse perinatal outcomes, including for example, premature delivery and low birthweight [17], in addition to a range of negative child outcomes that can persist into late adolescence, including an increased risk of child behaviour problems [18].

**Early pre- and postnatal experiences in the context of COVID-19 and associated societal restrictions**

The limited evidence on the impact of the pandemic and lockdowns on postnatal depression, suggest a similar picture, with around half of mothers caring for babies born during 2020 in the UK, reporting feeling down, lonely and worried, with mental health symptoms exacerbated in mothers who travel to work, had a baby born prematurely or were from a lower-income household [19]. One Australian study that examined all online perinatal support forum posts related to COVID-19, from women between January 27 to May 12, 2020, showed that the content was predominantly negative, with around 63% being very or moderately negative. Negative words that were frequently used in the 831 posts included: "worried" (n=165, 19.9%), "risk" (n = 143, 17.2%), "anxiety" (n = 98, 11.8%), "concerns" (n = 74, 8.8%), and "stress" (n = 69, 8.3%) [20].

Anxiety and depression in the postnatal period have been shown to affect the development of the infant because of the impact on the mother's interactions with her baby. For example, depressed mothers have been shown to be less sensitively attuned to infants, and less affirming and more negating of their experiences with their infant [21]. Babies of depressed mothers can exhibit deficits in their interpersonal functioning, such as less affective sharing, lower rates of interactive behaviour, poorer concentration, increased negative responses with strangers, and reduced secure attachment at 12 and 18 months [22,23]. Children of these women are also 42% more likely to experience depression by age 16 [24].

There is now increasing evidence regarding the impact of the mother's bond with her unborn baby, including her mental representations of the baby in pregnancy, in terms of an association with their interactions postnatally [25] including the infant's attachment status [26]. However, there is currently limited evidence regarding the impact of pandemic-related social restrictions on this prenatal relationship. While a number of studies have found that the pandemic has had an impact on bonding with the baby in the postnatal period [27] and on breastfeeding [28], no studies to date have explicitly examined the impact on the parent-child interaction. Furthermore, concerns have been raised regarding infants limited exposure to infant peers or to diverse social partners other than household members or members of the public wearing masks, in terms of the impact on infant looking and communicative bids during social interactions [29]. Whilst ongoing research examines how the pandemic and the above-

For peer review only



For peer review only - http://bmjopen.bmj.com/site/about/guidelines.xhtml

mentioned points are affecting children aged 8-36 months, there remains a critical lack of study of development during the period of development referred to as the “baby blind-spot” (from pregnancy to 2 years old age) [29,30].

There remain more questions empirical answers at the present time regarding how ‘stay at home’ orders, lack of access to social support from family members and pandemic-specific stressors might have affected expectant parents and those caring for babies during the national lockdowns. Concerns have been raised about women at risk for domestic violence, without suitable shelter and higher incidence of unemployment due to their role in the retail, caregiving and hospitality workforce [31,32]. It is also important to take into consideration other mediating influences on infant development including poverty [33], maternal education [34], marital discord [35], single parenthood and ethnicity [36].

Overall, little is currently known about the impact of COVID-19 guidance and restrictions on the long-term development of the child. To address this gap in our knowledge, this study has two main aims: i) to examine how parent’s experiences of the social, medical, and financial changes during the pandemic have impacted pre and postnatal parental mental health and parent-infant social interaction; and (ii) to investigate the extent to which these COVID related changes in parental pre and postnatal mental health and social interaction are associated with fetal and infant development.

## STUDY DESIGN

The Covid in the Context of Pregnancy, Infancy and Parenting (CoCoPIP) study, is a national online survey being carried out in the UK, which was widely advertised from July 2020, that continues to actively recruit families for participation. The research comprises a mixed-methods study collecting data inclusive of both; a) validated physical and psychological assessments, and b) open-ended questions to allow the participant to elaborate on their experience in their own words. The CoCoPIP Study addresses four key hypotheses (H1-4) (see *Figure 1*).

**[Insert figure 1]**

### Eligibility criteria and recruitment strategies

Eligibility criteria for the study is expectant parents (at any stage of pregnancy) or parents of an infant between the ages of 0-6 months. Either parent can take part, with questionnaires being adapted to the parents’ status as mother or father. The study is open to parents who had a baby shortly before and during the first period of lockdown in the UK (23rd March 2020) as well as continuing to collect information from parents during the current, and future changes in COVID-related health and societal restrictions.


For peer review only - http://bmjopen.bmj.com/site/about/guidelines.xhtml

For optimal national representation across the UK, recruitment strategies include (i) targeting NHS antenatal classes and National Childbirth Trust (NCT) groups identified nationally, with an emphasis on areas of low socio-economic status (SES) using the government indices of multiple deprivation (IMD) and rural areas without access to NCT groups, (ii) partnering with NHS/National Institute for Health Research (NIHR) collaborative sites and charity and policy group partners (e.g., The Brazelton Centre UK, Centre for Health and the Public Interest) to widen knowledge of the survey, (iii) posting online via social media platforms (e.g., Twitter) and public sharing to facilitate snowball sampling and (iv) targeting populations experiencing increased local lockdown measures as, and when, COVID-19 rates and related policy change across the UK. Whilst recruitment efforts have been focused on the UK, the survey is currently open to all expectant and new families worldwide. Participation in the survey is incentivized using the offer of a chance to win a £100 digital gift card (on receipt participants are able to select from either an Amazon® or one4all® gift card). A prize is drawn for every 100 participants who complete the survey, giving a 1/100 chance of winning at each time point that they complete.

**Patient and Public Involvement**

The study was designed with input from the public, particularly pregnant and new parents who had an infant during the onset of the pandemic from March – May 2020. Input included aspects such as wording of questions and ease of completing questionnaires (both visually and in length). Throughout Results will be disseminated to study participants through various social media platforms that participants are given links to during initial recruitment and in subsequent follow-up correspondence.

**Power Calculation**

To ensure sufficient power for the study, statistical power calculations (G*Power adapted for regression) based on three outcomes, up to three predictors and four co-variables, estimated a minimum sample size for Hypothesis 1 of n = 400 (small effect, $f^2 = 0.02$). Statistical power calculations were based on a study of sociodemographic control variables, traumatic event impact scale and pregnancy-specific anxiety [37]. In the same manner, for Hypothesis 2, a minimum sample size of n = 800 (small effect, n = 400 infants x 2 postnatal timepoints) is required, based on ongoing analyses by our group on parent-infant social interaction data [38]. For Hypothesis 3 and 4, we will minimise data loss using post-hoc assignment of families to an accelerated longitudinal design - this requires a minimum sample size of n = 500 (small effect) using a study on acute disasters, parental mental health and infant development [39]. With timing and cross lag accounted for, and attrition rate of 30% - assuming current pattern of 80% of parents' consenting to be contacted again (as indicated by our pilot survey) – a minimum cohort sample of n = 1500 is required.

For peer review only



For peer review only - http://bmjopen.bmj.com/site/about/guidelines.xhtml

**Study Measures**

The online survey is logic-dependent and adaptive, only showing questions relevant to the parent's current situation (e.g., antenatal or with an infant of 2 months of age) in relation to the following six time points: the second and third trimester of pregnancy; infant aged 0-3 months, infant aged 3-6 months; and toddler aged 12- and 18-months. The following data is collected: (i) parental mental health and attitudes, (ii) healthcare access and support during pregnancy and birth, (iii), fetal physical development and infant social and cognitive development, (iv) direct impact of COVID-19 on daily lives and lastly (v) developmental outcomes in infants born during the pandemic. *Table 1* provides an overview of the measures used timepoints and *Table 2* provides a detailed summary of the measures used within the survey.

**[Insert figure 2]**

**Follow-ups and reminders**

Participants are invited to take part in a follow-up survey at the end of the initial survey. Where they consent to this, they are contacted via email containing a link to the separate online survey. The follow-up survey has been condensed to include follow-up questionnaires only (*see Table 1* and *Figure 2* for participant follow-up flowchart). The appropriate time for follow-up is calculated based on the ages (infant or fetal gestation) provided by the participant at initial recruitment (see *Figure 2* for project timeline).

For peer review only

| | Timepoint | | | | | |
|---|---|---|---|---|---|---|
| | Pregnancy | | Infant | | Toddler | |
| | 1 | 2 | 3 | 4 | 5 | 6 |
| **Section A: Consent and participant background information** | | | | | | |
| Consent[a] | x | x | x | x | | |
| Demographics[b] | x | x | x | x | | |
| Income and employment status | x | x | x | x | | |
| **Section B: Pregnancy measures** | | | | | | |
| Fetal growth measures and pregnancy | x[c] | x[d] | | | | |
| Health care support and access | x | x | x | x | | |
| Antenatal Emotional Attachment Scale (AEAS) | x | x | | | | |
| Pregnancy Related Anxiety Questionnaire Revised (PRAQ-R) | x | x | | | | |
| **Section C: Infant birth and development measures** | | | | | | |
| Birth information[e] | | | x | x | | |
| Infant Behaviour Questionnaire (IBQ) | | | | x | x | |
| Infant Toddler Sensory Profile (ITSP) | | | x | x | x | |
| Infant related anxiety | | | x | x | | |
| Face-to-face interaction index | | | x | x | | |
| **Section D: Toddler Development measures** | | | | | | |
| Ages and Stages | | | | | x | |
| Oxford CDI | | | | | x | |
| Q-CHAT | | | | | | x |
| Vineland parent and caregiver form | | | | | | x |
| **Section E: Parental mental health and support measures** | | | | | | |
| State Trait Anxiety Index – State (STAI-S) | x | x | x | x | x | x |
| Caregiving, social interaction and support questionnaire | | | x | x | | |
| Stressful life events questionnaire | | | | | x | x |
| **Section F: Parenting and family measures** | | | | | | |
| Parenting reflective functioning questionnaire (PRFQ) | | | | | x | |
| Comprehensive early childhood parenting questionnaire (CECPAQ) | | | | | x | |
| **Section G: COVID Impact** | | | | | | |
| COVID situational influences | x | x | x | x | | |
| COVID health report | x | x | x | x | | |
| COVID concern and event impact scale | x | x | x | x | | |
| Social distance impacts | x | x | x | x | | |
| Vaccine | x | x | x | x | | |
| Difficulties in Emotion Regulation Scale (DERS) | | | | | x | |

[a-b] Participants will only be asked to complete this section once, when they initially join the study. The study can be joined at any timepoint. Those eligible will be asked if they wish to participate longitudinally.

[c] only physical questions in relation to 2nd trimester scan.

[d] only physical questions in relation to 3rd trimester scan.

[e] will only be asked to complete this section once

*Table 1: A summary of assessments and questionnaires used separated by time point.*



For peer review only - http://bmjopen.bmj.com/site/about/guidelines.xhtml

| Measure (items) | Description | Scale | Example item |
|---|---|---|---|
| **COVID-19 items** | | | |
| Situational influences | Assessing adjustment and adherence to government guidelines. | | 'When did you modify your routine to adhere to the social distancing guidance?' |
| Health report | Assessing risk for COVID exposure and symptoms of parents, partner and/or social network. As well as self-isolation behaviours and support (if applicable) | | 'Have you been diagnosed with COVID?' 'How supported did you feel during self-isolation?' |
| COVID Impact (7 items) | How COVID-19 has impacted themselves and their household (e.g., physical health, disruption to routine). | Five-point scale from 'not hard at all, copying well' to 'unbearable, not coping well' | |
| COVID Concern (10 items) | | Six-point scale from 'not at all' to 'all the time' | 'I am not worried about COVID-19' |
| Event Impact Scale-Revised [40] (20 items) | Assessing the distress experience as a result of traumatic events | Five-point scale from 'extremely' to 'not at all' | 'I felt irritable and angry' |
| Social distance impacts | Assessing participants attitudes and experience during COVID-19. | Five-point scale from 'not at all' to 'very true' | 'I spend my leisure time more intentionally' |
| Vaccines | Assessing access to and attitude towards a vaccine and potential changes in social behaviour in relation to a vaccine. | | 'Are you currently eligible for the COVID-19 vaccine?' / 'Could you tell us how and/or why getting vaccinated has changed the way you socialise?' |
| **Parental information** | | | |
| State-Trait Anxiety state (STAI-S) [41] (20 items) | Assessing the presence and severity of an individual's state-based anxiety (i.e., anxiety in response to what is happening right now). | Four-point scale from 'not at all' to 'very much so' | 'I feel strained' |
| Caregiving support | Questions assessing remote and in person availability of household members, partners, and social network of families | | |
| Home schooling | Exploring the additional demands on parents in the home and support available for home schooling. | | 'Could you describe your experience of home schooling during your pregnancy or as a new parent?' |
| Stress Life Inventory [42] (43 items) | Assessing the number of stressful life events experienced by the family over a 12-month period | Items rates 'yes' or 'no' | 'Being fired at work' |


For peer review only - http://bmjopen.bmj.com/site/about/guidelines.xhtml

| | | | |
|---|---|---|---|
| Pregnancy related anxiety questionnaire (PRAQ) [43] (20 items)* | Assessing pregnancy-specific anxiety in women during the antenatal period. | Five-point scale from ‘not at all’ to ‘very much’ | ‘I think that after birth my child will be in poor health’ / ‘I think about creating a virus-free home for my child’ |
| **Pregnancy information** | | | |
| Healthcare support and access | Exploring parental experiences of their healthcare support and care, and access to antenatal routine care during the pandemic | | ‘Do you feel comfortable attending your pregnancy appointments?’/ ‘Could you tell us about the support from your healthcare provides during your pregnancy’ |
| Antenatal Emotional Attachment Scale (AEAS) - Maternal [44] (19 items) | Questions asking expectant parents about their thoughts and feelings over the last two-week period about their baby | Responses on a varying five-point scale (e.g., ‘almost all the time’ to ‘not at all’ and ‘very positive’ to ‘very negative’) | ‘I think of the developing baby as mostly as;’ |
| Antenatal Emotional Attachment Scale (AEAS) – Paternal [44] (16 items) | | | ‘I have found myself touching, or rubbing with my hand, the outside of my partners stomach where the baby is:’ |
| **Toddler outcomes** | | | |
| Infant Behaviour Questionnaire (IBQ) – short form [45] (36 items) | Measures the dimensions of temperament in children younger than 12 months of age | Eight-point scale from ‘never’ to ‘always’ (including option for ‘does not apply’) | ‘When tired, how often did your baby show distress?’ |
| Infant/Toddler Sensory Profile (ITSP) [46] (36 items) | A caregiver report on how responsive, sensitive, avoidant and alert their babies are when presented with stimuli visual, auditory, tactile and vestibular modalities | Six-point scale from ‘almost always’ to ‘almost never’ (including an option for ‘does not apply’) | ‘My child is active throughout the day’ |
| Ages and Stages (ASQ-3) [47] (39 items) | A developmental screening tool assessing a child’s developmental progress in six domains (i.e., communication, gross motor, fine motor, problem solving and personal-social development). | Three-point scale from ‘yes’ to ‘not yet’ | ‘When your baby wants something, does he tell you by pointing to it?’ |
| Oxford Communication Development Inventory – 100 words (CDI) [48] | A UK-adapted measure of toddler language development | Two-point scale; ‘understands’ and ‘understands and says’ | ‘car’ |
| Quantitative Checklist for Autism in Toddlers (Q-CHAT) [49] (25 items) | A quantitative measure of early autistic traits in toddlers | Responses given on a varying five-point scale (e.g., ‘many times a day to never’ and ‘always’ to ‘never’) | ‘How easy is it for your child to adapt when his/her routine changes or when things are out of their usual place?’ |



For peer review only - http://bmjopen.bmj.com/site/about/guidelines.xhtml

| | | | |
|---|---|---|---|
| Vineland parent and caregiver form [50] | A number of questions about the home environment and family-life behaviour | Three-point scale from "usually or often' to 'never' | 'When you look or point at something, looks in that direction.' |
| **Parenting, family and home environment** | | | |
| Household space and environment | Assessing access to outdoor/indoor space and technology | | 'Do you currently have access to outdoor space you feel safe spending time in?' / 'In your home, how many mobile phones are presently in use?' |
| Parent-infant attachment | Parental description of relationship with infant (imagined and actual) | | 'Could you tell us a little about what you image your baby will be like and how the two of you will get along?' |
| Face-to-face interaction index | Exploring the frequency of face-to-face and distant interaction the infant has had since birth | | 'How often did you with/without your baby video chat with others?'/ 'How responsive is your baby to others during virtual interactions?' |
| Parenting reflective functioning questionnaire (PRFQ) [51] (18 item) | A short measure assessing parental reflective functioning or mentalising. | Responses are rated on a seven-point scale from 'strongly disagree' to 'strongly agree' | 'I always know why my child acts the way he or she does' |
| Comprehensive early childhood parenting questionnaire (CECPAQ) [52] (54 items) | A parent report measure assessing five domains of parenting (i.e., support, stimulation, structure, harsh discipline and positive measure). | Responses are rated on a six-point scale from 'never' to 'always' | 'when my child is having a hard time, I am able to help him/her. |
| Difficulties in Emotion Regulation Scale (DERS) [53] (19 items) | A self-report measure of subjective emotion ability | Responses are rated on a five-point scale from 'almost never' to 'almost always' | 'When I'm upset, I can still get things done' |
| Family demographics and socioeconomic questions | Parent and infant age or gestation of pregnancy; sex; ethnicity; location; parent and partner highest level of qualification; employment status and household income. | | 'what is your highest level of education?' |
| Postcode | used to derived indices of multiple deprivation. | | |

*several questions have been adapted for use during COVID-19

*Table 2: A detailed summary of measures used within the study.*


For peer review only - http://bmjopen.bmj.com/site/about/guidelines.xhtml

# DATA ANALYSIS PLAN

**Quality Control**

Ongoing quality control is evaluated bi-weekly. All data is checked for accuracy and invalid data is removed (e.g., one parent responded to the question "what is the gender of your child" as "clownfish" and completed no other question within the survey - this participant was removed from the dataset). Study data is collected and managed using Research Electronic Data Capture (REDCap®) tools hosted at the University of Cambridge [54]. REDCap® is a secure, web-based software platform designed to support data capture for research studies, providing 1) an intuitive interface for validated data capture; 2) audit trails for tracking data manipulation and export procedures; 3) automated export procedures for seamless data downloads to common statistical packages; and 4) procedures for data integration and interoperability with external sources. Personal data (e.g., caregiver DOB, email address) is stored securely within a password encrypted electronic databased isolated from the research data. Access to the data is fully audited to ensure data security is governed by a management team and in compliance with ethical guidelines.

**Analysis plan**

Overall, we aim to identify which stress-associated moderators (i.e., loss of income, COVID-19 illness, local access to ante/postnatal support) impact significantly on parental mental health, and in turn, infant development.

To address Hypothesis 1 (*see Figure 1*), a combination of quantitative and qualitative analyses will be undertaken. Structural equation modelling (SEM) and hypothesis-driven regressions will explore how multiple aspects of pre- and post-natal family support (social, financial and health) are associated with stress (maternal and paternal) and wider mental health. An inductive approach to data analysis will be undertaken [55] for the open-ended qualitative data, and we will use NVivo (QSR International Pty Ltd) to code the data. Following this, several approaches including thematic [56], sentiment and context content analysis will be undertaken using a natural language processing (NLP) approach [57] (machine learning) to identify forms of social, medical and financial support in relation to the valence of parental attitudes. Regression analyses will then be conducted to understand the directional relationship between resulting latent factors (qualitative responses and quantitative data) and parental mental health.

To address Hypothesis 2, regressions will be used to explore how COVID-19 related restrictions interact with the frequency and forms of social interaction (i.e., face-to-face, digital, at distance) that the infant has, and whether this varies across the first six months of life.


For peer review only - http://bmjopen.bmj.com/site/about/guidelines.xhtml

To address Hypothesis 3, regressions will be used to explore the influence of maternal mental health longitudinally on the developing offspring across pre- to post-natal life: from fetal (12-weeks/20 weeks gestational age) to 18 months of age. Standardised z-scores will be created from the collected fetal growth measurements (i.e., head circumference, femur length and abdominal circumference), which will then be transformed into a composite score accounting for gestational age and/or estimated fetal weight at time of scan to be used for analysis. Z-scores will also be computed and used where appropriate within the analysis (e.g., infant-toddler sensory profile).

To address Hypothesis 4, outputs from Hypothesis 1 and 2 (impact of COVID-19 on parental mental health and infant social interactions) will be explored in relation to longitudinal social and cognitive development (e.g., language, motor sensory and the early emergence of developmental conditions) of the infant/toddler across the 0-18 months of life using SEM, full information maximum likelihood to account for missingness, and regression modelling.

## CURRENT COHORT DESCRIPTION AND DEMOGRAPHICS

Initiated in July 2020, this study is ongoing with n = 1700 families currently enrolled (6th May 2021). Parents can consent to complete the questionnaire up to six times during pregnancy/parenting until their infants are 18 months of age. For those participants who contribute more than one time point (between antenatal and postnatal timepoints ≤ 6 months) an invitation is issued for a follow up to assess their toddler's development when aged 12 and 18 months (see *Figure 2* for study flow chart).

To date 1700 of families have participated in at least one time-point of the study, with 641 families joining at time points 1-2, 372 families at time-point 3, and 687 families at time-point 4 (see *Figure 2 and Table 1* for time-points). 61% of these families have consented to completing the subsequent follow-up sections of the study. To date 97.4% of respondents identify as mothers, 2.3% as fathers and 0.3% as another parent or caregiver, with the majority of participating families disclosing their ethnicity as white (89.2%). Those participating families who are from the UK have their household information (i.e., household income, location of participating families, index of multiple deprivation and respondent's education level) described in *Figure 3*.

**[Insert figure 3]**

# ETHICS AND DISSEMINATION

Ethics approval for the survey was given by the University of Cambridge, Psychology Research Ethics Committee (PREC) (PRE.2020.077). All respondents are required to be over the age of 18 years and give electronic informed consent. Caregivers agreeing to be followed-up longitudinally give consent at each timepoint and are made aware that their participation can be stopped at any time within the study.


For peer review only - http://bmjopen.bmj.com/site/about/guidelines.xhtml

Permissions have been obtained from participants to ensure anonymised data can be made available on open-source platforms.

A proactive dissemination pathway has been established from the outset. We will engage with policy stakeholders (health practitioners/Department of Health) and social media platforms to create discussion around this topic. Dissemination of findings will be via public forums (i.e., social media, media, collaborators family dissemination pathways) and at the national (i.e., NHS England/NHS Improvement, Royal College of Paediatrics and Child Health, Centre for Health and the Public Interest) and local (Cambridge Universities Hospitals NHS Foundation Trust) level. Data will continue to be disseminated throughout the period of the study to promote discussion and raise the profile of the population identified as being one of the most vulnerable and neglected during the pandemic.

To date, qualitative responses from the first five months of data collection have been analysed to explore parents experiences of being pregnant in relation to healthcare access during the pandemic [5]. This was conducted using thematic and sentiment analysis. The initial findings suggest that a range of adverse effects have been experienced by expectant parents in the UK relating to changes in antenatal support and healthcare appointments in response to governmental guidance with regard to social distancing. These findings point to an urgent need to better address the unique health care needs of each pregnant woman going forward.

## Data Sharing Plan

Questionnaires and study goals were made available upon request using the Open Science Foundation platform in July 2020 and made public at https://osf.io/m7zuw/ in August 2020. Study protocol, follow-up questionnaires and statistical analysis code will be uploaded and shared to facilitate data sharing and collaboration, in accordance with Research, Innovation and Science Policy Experts (RISE) EU principles [58]. Data collected will be uploaded to open-source platforms, for utilisation by other researchers and policy stakeholders. In addition, quarterly layman summaries of findings will be reported for parents and the general public on our website and social media platforms. Qualitative data generated and analysed during the study will not be made publicly available due to ethical and privacy restrictions, however researchers can submit a research proposal to the Data Sharing Management Committee to request access and collaboration. We will either make our datafile associated with each publication available on an opensource platform, following peer review and publication or 12 months after the completion of a follow-up timepoint.



For peer review only - http://bmjopen.bmj.com/site/about/guidelines.xhtml

**Acknowledgements:** We are extremely grateful to all those families who gave their time to participate and to Esther Adememo, Maddie Walton and Zahra Khan who worked on the CoCoPIP study during their undergraduate and master's studies at Cambridge.

**Author statement: Ezra Aydin:** Conceptualization, Methodology, Writing - Original Draft. **Staci M. Weiss:** Conceptualization, Methodology, Writing - Original Draft. **Kevin A. Glasgow:** Methodology, Visualisation, Writing - Review & Editing **Topun Austin:** Methodology, Supervision, Writing - Review & Editing. **Mark H. Johnson:** Supervision, Funding acquisition, Writing - Review & Editing. **Jane Barlow:** Supervision, Writing - Review & Editing. **Sarah Lloyd-Fox:** Conceptualization, Methodology, Supervision, Funding acquisition, Writing - Review & Editing.

**Funding source:** This research was funded by a Medical Research Council Programme Grant MR/T003057/1 to MJ, and an UKRI Future Leaders fellowship (MRC grant MR/S018425/1) to SLF. The views expressed are those of the authors and not necessarily those of the MRC or the UKRI.

The NIHR Cambridge Biomedical Research Centre (BRC) is a partnership between Cambridge University Hospitals NHS Foundation Trust and the University of Cambridge, funded by the National Institute for Health Research (NIHR), T.A. is supported by the NIHR Cambridge Biomedical Research Centre (BRC). TA is also supported by the NIHR Brain Injury MedTech Co-operative. The views expressed are those of the author(s) and not necessarily those of the NIHR or the Department of Health and Social Care.

**Disclosure Statement:** The authors have no potential conflicts of interest to disclose.

For peer review only

**Figure Legends**

*Figure 1: Covid in the Context of Pregnancy, Infancy and Parenting (CoCoPIP) study four key hypotheses.*

A participant can join the study at any of the above 3 underlined timepoints

*Participants are only followed up at these two timepoints if they have participated in at least two previous timepoints.

** Projected participant follow-up completion dates.

*Figure 2: Flow diagram of Covid in the Context of Pregnancy, Infancy and Parenting (CoCoPIP) study follow up participation.*

*Figure 3: Bubble map depicting spread of participations location in the UK (if postcode was provided) with respondent's education level, Index of Multiple Deprivation (IMD) and household income breakdown reported on the right.*

For peer review only



For peer review only - http://bmjopen.bmj.com/site/about/guidelines.xhtml

**REFERENCES**


1 Laplante DP, Brunet A, Schmitz N, *et al.* Project Ice Storm: Prenatal Maternal Stress Affects Cognitive and Linguistic Functioning in 5½-Year-Old Children. *J Am Acad Child Adolesc Psychiatry* 2008;**47**:1063–72. doi:10.1097/CHI.0b013e31817eec80

2 King S, Dancause K, Turcotte-Tremblay A-M, *et al.* Using Natural Disasters to Study the Effects of Prenatal Maternal Stress on Child Health and Development: Natural Disasters and Prenatal Maternal Stress. *Birth Defects Res Part C Embryo Today Rev* 2012;**96**:273–88. doi:10.1002/bdrc.21026

3 Nelson III CA, Gabard-Durnam LJ. Early adversity and critical periods: neurodevelopmental consequences of violating the expectable environment. *Trends Neurosci* 2020;**43**:133–43.

4 Davenport MH, Meyer S, Meah VL, *et al.* Moms are not ok: COVID-19 and maternal mental health. *Front Glob Womens Health* 2020;**1**:1.

5 Aydin E, Glasgow KA, Weiss S, *et al.* Expectant parents' perceptions of healthcare and support during COVID-19 in the UK: A thematic analysis. Public and Global Health 2021. doi:10.1101/2021.04.14.21255490

6 Barlow J, Bach-Mortensen A, Homonchuk O, *et al.* The impact of the COVID-19 pandemic on services from pregnancy through age 5 years for families who are high risk of poor outcomes or who have complex social needs. 2020.

7 Pierce M, Hope H, Ford T, *et al.* Mental health before and during the COVID-19 pandemic: a longitudinal probability sample survey of the UK population. *Lancet Psychiatry* 2020;**7**:883–92. doi:10.1016/S2215-0366(20)30308-4

8 Shah SMA, Mohammad D, Qureshi MFH, *et al.* Prevalence, Psychological Responses and Associated Correlates of Depression, Anxiety and Stress in a Global Population, During the Coronavirus Disease (COVID-19) Pandemic. *Community Ment Health J* 2021;**57**:101–10. doi:10.1007/s10597-020-00728-y

9 Davis N. NHS trusts begin suspending home births due to coronavirus. 2020.

10 NHS. Supporting Pregnant women using maternity service during the coronavirus pandemic: Actions for NHS providers. 2020.

11 Sherwood H. Midwife shortage doubles as NHS staff diverted to tend Covid-19 patients. 2020.

12 Khalil A, Von Dadelszen P, Draycott T, *et al.* Change in the incidence of stillbirth and preterm delivery during the COVID-19 pandemic. *Jama* 2020;**324**:705–6.

13 Preis H, Mahaffey B, Heiselman C, *et al.* Vulnerability and resilience to pandemic-related stress among U.S. women pregnant at the start of the COVID-19 pandemic. *Soc Sci Med* 2020;**266**:113348. doi:10.1016/j.socscimed.2020.113348

14 Moyer CA, Compton SD, Kaselitz E, *et al.* Pregnancy-related anxiety during COVID-19: a nationwide survey of 2740 pregnant women. *Arch Womens Ment Health* 2020;**23**:757–65. doi:10.1007/s00737-020-01073-5

15 Lebel C, MacKinnon A, Bagshawe M, *et al.* Elevated depression and anxiety symptoms among pregnant individuals during the COVID-19 pandemic. *J Affect Disord* 2020;**277**:5–13. doi:10.1016/j.jad.2020.07.126


For peer review only



For peer review only - http://bmjopen.bmj.com/site/about/guidelines.xhtml

16 Berthelot N, Lemieux R, Garon-Bissonnette J, *et al.* Uptrend in distress and psychiatric symptomatology in pregnant women during the coronavirus disease 2019 pandemic. *Acta Obstet Gynecol Scand* 2020;**99**:848–55. doi:10.1111/aogs.13925

17 Grigoriadis S, Graves L, Peer M, *et al.* Maternal Anxiety During Pregnancy and the Association With Adverse Perinatal Outcomes: Systematic Review and Meta-Analysis. *J Clin Psychiatry* 2018;**79**. doi:10.4088/JCP.17r12011

18 Stein A, Pearson RM, Goodman SH, *et al.* Effects of perinatal mental disorders on the fetus and child. *The Lancet* 2014;**384**:1800–19. doi:10.1016/S0140-6736(14)61277-0

19 Dib S, Rougeaux E, Vázquez-Vázquez A, *et al.* Maternal mental health and coping during the COVID-19 lockdown in the UK: Data from the COVID-19 New Mum Study. *Int J Gynecol Obstet* 2020;**151**:407–14. doi:10.1002/ijgo.13397

20 Chivers BR, Garad RM, Boyle JA, *et al.* Perinatal Distress During COVID-19: Thematic Analysis of an Online Parenting Forum. *J Med Internet Res* 2020;**22**:e22002. doi:10.2196/22002

21 Murray L, Fiori-Cowley A, Hooper R, *et al.* The Impact of Postnatal Depression and Associated Adversity on Early Mother-Infant Interactions and Later Infant Outcome. *Child Dev* 1996;**67**:2512. doi:10.2307/1131637

22 Murray L. The Impact of Postnatal Depression on Infant Development. *J Child Psychol Psychiatry* 1992;**33**:543–61. doi:10.1111/j.1469-7610.1992.tb00890.x

23 Stein A, Gath DH, Bucher J, *et al.* The Relationship between Post-natal Depression and Mother–Child Interaction. *Br J Psychiatry* 1991;**158**:46–52. doi:10.1192/bjp.158.1.46

24 Murray L, Arteche A, Fearon P, *et al.* Maternal Postnatal Depression and the Development of Depression in Offspring Up to 16 Years of Age. *J Am Acad Child Adolesc Psychiatry* 2011;**50**:460–70. doi:10.1016/j.jaac.2011.02.001

25 Foley S, Hughes C. Great expectations? Do mothers' and fathers' prenatal thoughts and feelings about the infant predict parent-infant interaction quality? A meta-analytic review. *Dev Rev* 2018;**48**:40–54. doi:10.1016/j.dr.2018.03.007

26 Barlow J, Underdown A. Child maltreatment during infancy: atypical parent–infant relationships. *Paediatr Child Health* 2018;**28**:114–9. doi:10.1016/j.paed.2017.11.001

27 Mayopoulos GA, Ein-Dor T, Dishy GA, *et al.* COVID-19 is associated with traumatic childbirth and subsequent mother-infant bonding problems. *J Affect Disord* 2021;**282**:122–5. doi:10.1016/j.jad.2020.12.101

28 Brown A, Shenker N. Experiences of breastfeeding during COVID-19: Lessons for future practical and emotional support. *Matern Child Nutr* 2021;**17**. doi:10.1111/mcn.13088

29 Bhopal S, Fearon P. Pandemic babies: how COVID-19 has affected child development. 2021.https://theconversation.com/pandemic-babies-how-covid-19-has-affected-child-development-155903

30 Reed J, Parish N. Working for babies: Lockdown lessons from local systems. 2021. https://parentinfantfoundation.org.uk/wp-content/uploads/2021/01/210115-F1001D-Working-for-Babies-Report-FINAL-v1.0-compressed.pdf

For peer review only

31 European Parliament. Directorate General for Internal Policies of the Union. *The gendered impact of the Covid-19 crisis and post-crisis period.* LU: : Publications Office 2020. https://data.europa.eu/doi/10.2861/393602 (accessed 15 Mar 2021).

32 Cook R, Grimshaw D. A gendered lens on COVID-19 employment and social policies in Europe. *Eur Soc* 2021;**23**:S215–27. doi:10.1080/14616696.2020.1822538

33 Petterson SM, Albers AB. Effects of Poverty and Maternal Depression on Early Child Development. *Child Dev* 2001;**72**:1794–813. doi:10.1111/1467-8624.00379

34 Pearson RM, Evans J, Kounali D, *et al.* Maternal Depression During Pregnancy and the Postnatal Period: Risks and Possible Mechanisms for Offspring Depression at Age 18 Years. *JAMA Psychiatry* 2013;**70**:1312. doi:10.1001/jamapsychiatry.2013.2163

35 Dawson G, Ashman SB, Panagiotides H, *et al.* Preschool Outcomes of Children of Depressed Mothers: Role of Maternal Behavior, Contextual Risk, and Children's Brain Activity. *Child Dev* 2003;**74**:1158–75. doi:10.1111/1467-8624.00599

36 Goodman SH, Rouse MH, Connell AM, *et al.* Maternal Depression and Child Psychopathology: A Meta-Analytic Review. *Clin Child Fam Psychol Rev* 2011;**14**:1–27. doi:10.1007/s10567-010-0080-1

37 Buss C, Davis EP, Hobel CJ, *et al.* Maternal pregnancy-specific anxiety is associated with child executive function at 6–9 years age. *Stress* 2011;**14**:665–76. doi:10.3109/10253890.2011.623250

38 Thomas JC, Letourneau N, Campbell TS, *et al.* Developmental origins of infant emotion regulation: Mediation by temperamental negativity and moderation by maternal sensitivity. *Dev Psychol* 2017;**53**:611–28. doi:10.1037/dev0000279

39 Letcher P, Greenwood CJ, Romaniuk H, *et al.* Adolescent and young adult mental health problems and infant offspring behavior: Findings from a prospective intergenerational cohort study. *J Affect Disord* 2020;**272**:521–8. doi:10.1016/j.jad.2020.03.101

40 Weiss DS, Marmar C, Wilson JP, *et al.* Assessing psychological trauma and PTSD. *Impact Events Scale—Revised* 1997;**19**:399–411.

41 Spielberger CD. State-Trait anxiety inventory. *Corsini Encycl Psychol* 2010;:1–1.

42 Noone PA. The Holmes–Rahe Stress Inventory. *Occup Med* 2017;**67**:581–2. doi:10.1093/occmed/kqx099

43 Brunton RJ, Dryer R, Saliba A, *et al.* The initial development of the Pregnancy-related Anxiety Scale. *Women Birth* 2019;**32**:e118–30. doi:10.1016/j.wombi.2018.05.004

44 Condon JT. The assessment of antenatal emotional attachment: development of a questionnaire instrument. *Br J Med Psychol* 1993;**66 ( Pt 2)**:167–83. doi:10.1111/j.2044-8341.1993.tb01739.x

45 Peterson ER, Waldie KE, Mohal J, *et al.* Infant Behavior Questionnaire-Revised Very Short Form: A New Factor Structure's Associations With Parenting Perceptions and Child Language Outcomes. *J Pers Assess* 2017;**99**:561–73. doi:10.1080/00223891.2017.1287709

46 Daniels DB, Dunn WW. Initial Development of the Infant/Toddler Sensory Profile. *J Early Interv* 2002;**25**:27–41. doi:10.1177/105381510202500104

47 Houser J. Ages And Stages. *Child Fam Serv Update* 2004;:3–3. doi:10.1037/e597192011-002

For peer review only


For peer review only - http://bmjopen.bmj.com/site/about/guidelines.xhtml

48 Hamilton a, Plunkett K, Schafer G. Infant vocabulary development assessed with a British communicative development inventory. *J Child Lang* 2000;**27**:689–705. doi:10.1017/S0305000900004414

49 Allison C, Baron-Cohen S, Wheelwright S, *et al.* The Q-CHAT (Quantitative CHecklist for Autism in Toddlers): A normally distributed quantitative measure of autistic traits at 18-24 months of age: Preliminary report. *J Autism Dev Disord* 2008;**38**:1414–25. doi:10.1007/s10803-007-0509-7

50 Burger-Caplan R, Saulnier CA, Sparrow SS. Vineland Adaptive Behavior Scales. In: Kreutzer JS, DeLuca J, Caplan B, eds. *Encyclopedia of Clinical Neuropsychology*. Cham: : Springer International Publishing 2018. 3597–601. doi:10.1007/978-3-319-57111-9_1602

51 Luyten P, Mayes LC, Nijssens L, *et al.* The parental reflective functioning questionnaire: Development and preliminary validation. *PLOS ONE* 2017;**12**:e0176218. doi:10.1371/journal.pone.0176218

52 Verhoeven M, Deković M, Bodden D, *et al.* Development and initial validation of the comprehensive early childhood parenting questionnaire (CECPAQ) for parents of 1–4 year-olds. *Eur J Dev Psychol* 2017;**14**:233–47. doi:10.1080/17405629.2016.1182017

53 Bjureberg J, Ljótsson B, Tull MT, *et al.* Development and Validation of a Brief Version of the Difficulties in Emotion Regulation Scale: The DERS-16. *J Psychopathol Behav Assess* 2016;**38**:284–96. doi:10.1007/s10862-015-9514-x

54 Harris PA, Taylor R, Thielke R, *et al.* Research electronic data capture (REDCap)—A metadata-driven methodology and workflow process for providing translational research informatics support. *J Biomed Inform* 2009;**42**:377–81. doi:10.1016/j.jbi.2008.08.010

55 Gerrish K, Lathlean J, editors. *The research process in nursing*. Seventh edition. Chichester, West Sussex, UK ; Malden, MA: : John Wiley & Sons Inc 2015.

56 Pope C, Ziebland S, Mays N. Qualitative research in health care. Analysing qualitative data. *BMJ* 2000;**320**:114–6. doi:10.1136/bmj.320.7227.114

57 Leeson W, Resnick A, Alexander D, *et al.* Natural Language Processing (NLP) in Qualitative Public Health Research: A Proof of Concept Study. *Int J Qual Methods* 2019;**18**:160940691988702. doi:10.1177/1609406919887021

58 European Commission. Directorate General for Research and Innovation. *Europe's future: open innovation, open science, open to the world : reflections of the Research, Innovation and Science Policy Experts (RISE) High Level Group.* LU: : Publications Office 2017. https://data.europa.eu/doi/10.2777/348700 (accessed 20 May 2021).


For peer review only



For peer review only - http://bmjopen.bmj.com/site/about/guidelines.xhtml

For peer review only


For peer review only - http://bmjopen.bmj.com/site/about/guidelines.xhtml

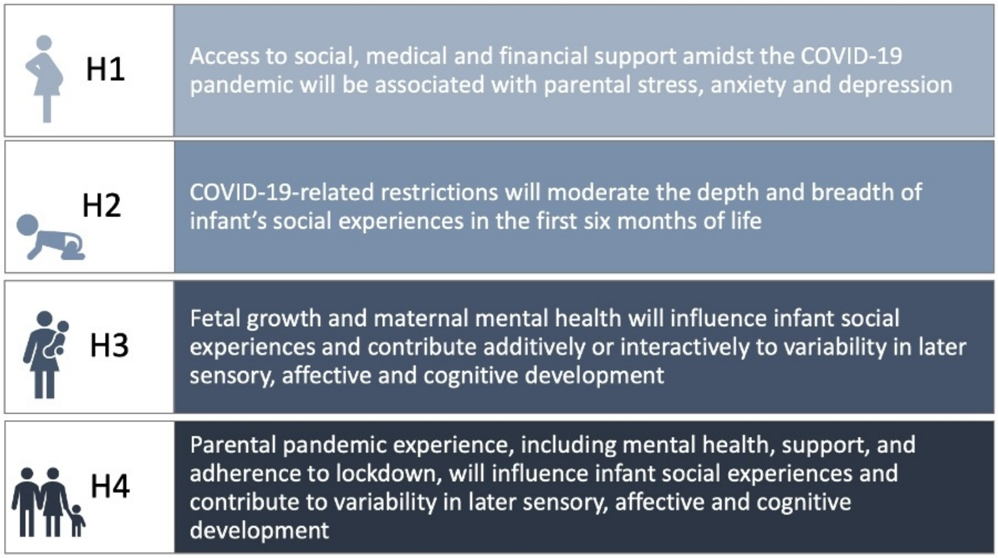


Figure 1: Covid in the Context of Pregnancy, Infancy and Parenting (CoCoPIP) study four key hypotheses.

159x89mm (144 x 144 DPI)

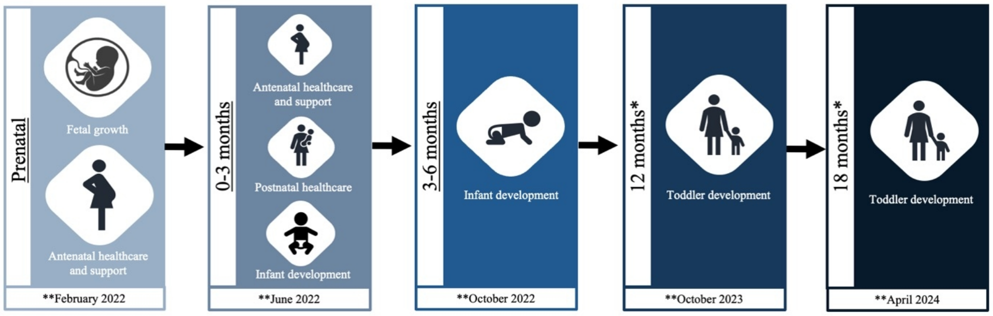


A participant can join the study at any of the above 3 underlined timepoints
*Participants are only followed up at these two timepoints if they have participated in at least two previous timepoints.
** Projected participant follow-up completion dates.
Figure 2: Flow diagram of Covid in the Context of Pregnancy, Infancy and Parenting (CoCoPIP) study follow up participation.

159x51mm (220 x 220 DPI)

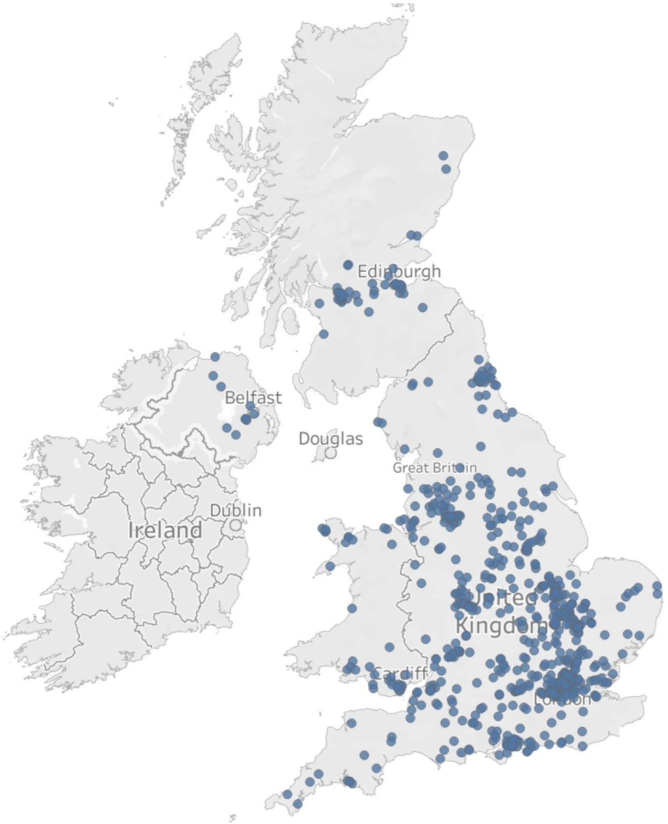


**Respondents Education Level**

| | n (%) |
|---|---|
| School ≤ 16 years old | 48 (3.2) |
| College ≤ 18 years old | 242 (16.3) |
| University | 1184 (80.5) |
| Undisclosed | 7 (0.5) |

**Index of Multiple Deprivation (IMD)**

| | n (%) |
|---|---|
| Most Deprived | 10 (2.3) |
| 2 | 20 (4.65 |
| 3 | 37 (8.4) |
| 4 | 43 (9.7) |
| 5 | 39 (8.8) |
| 6 | 62 (14.0) |
| 7 | 56 (12.6) |
| 8 | 54 (12.2) |
| 9 | 44 (9.9) |
| Least Deprived | 78 (17.6) |

**Participant report of household income**

| | n (%) |
|---|---|
| Less than 5,000 | 9 (0.6) |
| 5,001 – 10,000 | 15 (1.0) |
| 10,001 – 20,000 | 39 (2.7) |
| 20,001 – 30,000 | 92 (6.3) |
| 30,001 – 50,000 | 296 (20.4) |
| 50,001 – 75,000 | 383 (26.4) |
| 75,001 – 100,000 | 275 (19.0) |
| 100,001 – 150,000 | 161 (11.1) |
| 150,001- 200,000 | 48 (3.3) |
| 200,001 – 250,000 | 7 (0.5) |
| Greater than 250,001 | 11 (0.8) |
| Undisclosed | 113 (7.8) |

Figure 3: Bubble map depicting spread of participations location in the UK (if postcode was provided) with respondent's education level, Index of Multiple Deprivation (IMD) and household income breakdown reported on the right.

149x144mm (300 x 300 DPI)

**STROBE 2007 (v4) Statement—Checklist of items that should be included in reports of *cohort studies***

| Section/Topic | Item # | Recommendation | Reported on page # |
|---|---|---|---|
| **Title and abstract** | 1 | (*a*) Indicate the study's design with a commonly used term in the title or the abstract | 1-2 |
| | | (*b*) Provide in the abstract an informative and balanced summary of what was done and what was found | 2 |
| **Introduction** | | | |
| Background/rationale | 2 | Explain the scientific background and rationale for the investigation being reported | 4-7 |
| Objectives | 3 | State specific objectives, including any prespecified hypotheses | 7-8 |
| **Methods** | | | |
| Study design | 4 | Present key elements of study design early in the paper | 7-8 |
| Setting | 5 | Describe the setting, locations, and relevant dates, including periods of recruitment, exposure, follow-up, and data collection | 8-10 |
| Participants | 6 | (*a*) Give the eligibility criteria, and the sources and methods of selection of participants. Describe methods of follow-up | 8-10 |
| | | (*b*) For matched studies, give matching criteria and number of exposed and unexposed | n/a |
| Variables | 7 | Clearly define all outcomes, exposures, predictors, potential confounders, and effect modifiers. Give diagnostic criteria, if applicable | 11-16 |
| Data sources/ measurement | 8* | For each variable of interest, give sources of data and details of methods of assessment (measurement). Describe comparability of assessment methods if there is more than one group | 16 |
| Bias | 9 | Describe any efforts to address potential sources of bias | 8 |
| Study size | 10 | Explain how the study size was arrived at | 9 |
| Quantitative variables | 11 | Explain how quantitative variables were handled in the analyses. If applicable, describe which groupings were chosen and why | 15-16 |
| Statistical methods | 12 | (*a*) Describe all statistical methods, including those used to control for confounding | 15-16 |
| | | (*b*) Describe any methods used to examine subgroups and interactions | n/a |
| | | (*c*) Explain how missing data were addressed | n/a |
| | | (*d*) If applicable, explain how loss to follow-up was addressed | n/a |
| | | (*e*) Describe any sensitivity analyses | n/a |
| **Results** | | | |

For peer review only

| | | | |
|---|---|---|---|
| Participants | 13* | (a) Report numbers of individuals at each stage of study—eg numbers potentially eligible, examined for eligibility, confirmed eligible, included in the study, completing follow-up, and analysed | 16 |
| | | (b) Give reasons for non-participation at each stage | n/a |
| | | (c) Consider use of a flow diagram | 10 |
| Descriptive data | 14* | (a) Give characteristics of study participants (eg demographic, clinical, social) and information on exposures and potential confounders | 16 |
| | | (b) Indicate number of participants with missing data for each variable of interest | n/a |
| | | (c) Summarise follow-up time (eg, average and total amount) | 10 |
| Outcome data | 15* | Report numbers of outcome events or summary measures over time | 11 |
| Main results | 16 | (*a*) Give unadjusted estimates and, if applicable, confounder-adjusted estimates and their precision (eg, 95% confidence interval). Make clear which confounders were adjusted for and why they were included | n/a |
| | | (*b*) Report category boundaries when continuous variables were categorized | n/a |
| | | (*c*) If relevant, consider translating estimates of relative risk into absolute risk for a meaningful time period | n/a |
| Other analyses | 17 | Report other analyses done—eg analyses of subgroups and interactions, and sensitivity analyses | n/a |
| **Discussion** | | | |
| Key results | 18 | Summarise key results with reference to study objectives | n/a |
| **Limitations** | | | |
| Interpretation | 20 | Give a cautious overall interpretation of results considering objectives, limitations, multiplicity of analyses, results from similar studies, and other relevant evidence | n/a |
| Generalisability | 21 | Discuss the generalisability (external validity) of the study results | n/a |
| **Other information** | | | |
| Funding | 22 | Give the source of funding and the role of the funders for the present study and, if applicable, for the original study on which the present article is based | 23 |

*Give information separately for cases and controls in case-control studies and, if applicable, for exposed and unexposed groups in cohort and cross-sectional studies.

**Note:** An Explanation and Elaboration article discusses each checklist item and gives methodological background and published examples of transparent reporting. The STROBE checklist is best used in conjunction with this article (freely available on the Web sites of PLoS Medicine at http://www.plosmedicine.org/, Annals of Internal Medicine at http://www.annals.org/, and Epidemiology at http://www.epidem.com/). Information on the STROBE Initiative is available at www.strobe-statement.org.

For peer review only

BMJ Open

# The COVID in the Context of Pregnancy, Infancy and Parenting (CoCoPIP) Study: protocol for a longitudinal study of parental mental health, social interactions, physical growth, and cognitive development of infants during the pandemic.



| | |
|---|---|
| Journal: | *BMJ Open* |
| Manuscript ID | bmjopen-2021-053800.R1 |
| Article Type: | Protocol |
| Date Submitted by the Author: | 19-Jan-2022 |



| | |
|---|---|
| Complete List of Authors: | Aydin, Ezra; University of Cambridge, Department of Psychology<br>Weiss, Staci; University of Cambridge, Department of Psychology<br>Glasgow, Kevin; University of Cambridge, Department of Education<br>Barlow, Jane; University of Oxford, Department of Social Policy<br>Austin, Topun; Rosie Hospital; NIHR Cambridge Biomedical Research Centre<br>Johnson, Mark H.; University of Cambridge, Department of Psychology<br>Lloyd-Fox, Sarah; University of Cambridge, Department of Psychology |



| | |
|---|---|
| <b>Primary Subject Heading</b>: | Public health |
| Secondary Subject Heading: | Health policy, Qualitative research |
| Keywords: | COVID-19, QUALITATIVE RESEARCH, Fetal medicine < OBSTETRICS |
| | |


SCHOLARONE™
Manuscripts

# The COVID in the Context of Pregnancy, Infancy and Parenting (CoCoPIP) Study: protocol for a longitudinal study of parental mental health, social interactions, physical growth, and cognitive development of infants during the pandemic.

Ezra Aydin, PhD[1,2*], Staci M. Weiss, PhD[1*], Kevin A. Glasgow, MPhil[3], Jane Barlow, PhD[4], Topun Austin, MD[5,6], Mark H. Johnson, PhD[1,7] and Sarah Lloyd-Fox, PhD[1]

[1]Department of Psychology, University of Cambridge, UK.

[2]Department of Psychiatry, Columbia University, USA.

[3]Department of Education, University of Cambridge, UK.

[4]Centre for Evidence-Based Intervention, Department of Social Policy and Intervention, University of Oxford, UK.

[5]The Rosie Hospital, Cambridge University Hospitals NHS Foundation Trust, Cambridge, UK.

[6]NIHR Cambridge Biomedical Research Centre, Cambridge, UK.

[7]Centre for Brain & Cognitive Development, Birkbeck, University of London

*Joint first author.

Correspondence to: Ezra Aydin, Department of Psychology, University of Cambridge, Downing Street, Cambridge, CB2 3EB, UK. Email: ea420@cam.ac.uk Telephone: +44 (0) 1223 465 222

Submission to: BMJ Open

Word limit: 4000

Current word count: 4596

Table count: 1

Figure count: 3

For peer review only

# ABSTRACT

**Introduction** While the secondary impact of the COVID pandemic on the psychological wellbeing of pregnant women and parents has become apparent over the past year, the impact of these changes on early social interactions, physical growth and cognitive development of their infants is unknown, as is the way in which a range of COVID-related changes have mediated this impact. This study (CoCoPIP) will investigate: (i) how parent's experiences of the social, medical, and financial changes during the pandemic have impacted pre and postnatal parental mental health and parent-infant social interaction; and (ii) the extent to which these COVID-related changes in parental pre and postnatal mental health and social interaction are associated with fetal and infant development.

**Methods and analysis** The CoCoPIP study is a national online survey initiated in July 2020. This ongoing study (n = 1700 families currently enrolled as of 6$^{th}$ May 2021) involves both quantitative and qualitative data being collected across pregnancy and infancy. It is designed to identify the longitudinal impact of the pandemic from pregnancy to two years of age as assessed using a range of parent- and self-report measures, with the aim of identifying if stress-associated moderators (i.e., loss of income, COVID-19 illness, access to ante/postnatal support) appear to impact parental mental health, and in turn, infant development. In addition, we aim to document individual differences in social and cognitive development in toddlers who were born during restrictions intended to mitigate COVID-19 spread (e.g., social distancing, national lockdowns).

**Ethics and dissemination** Ethical approval was given by the University of Cambridge, Psychology Research Ethics Committee (PREC) (PRE.2020.077). Findings will be made available via community engagement, public forums (e.g., social media,) and to national (e.g., NHS England) and local (Cambridge Universities Hospitals NHS Foundation Trust) healthcare partners. Results will be submitted for publication in peer-reviews journals.


For peer review only

***Strengths and Limitations of this study***

- This is a new cohort of families being followed from prenatal to postnatal (up to 18 months) during the COVID-19 pandemic.
- The study involves the collection of quantifiable parent-report data to identify the short- and long-term influences of the pandemic on key aspects of infant development.
- The study also has a range of open-ended questions for qualitative analysis aimed at exploring familial experiences in more detail.
- The data is being collected online and is therefore limited to self- and parent-report measures, with no direct assessment of child development and parental mental health.
- Although the sample of families being recruited are diverse in their indices of multiple deprivation (IMD) and geographic location, they may not be fully representative of the wider population.

For peer review only

## INTRODUCTION

The COVID-19 pandemic has resulted in an unprecedented challenge to existing medical, social, and economic institutions, raising the risk for exposure to adversity for families expecting or parenting babies akin to prior natural disasters, war, or other periods of hardship [1]. Infants born during periods of social disruption and disease are noted for more restricted intrauterine growth, smaller birth size, and higher lifetime incidence of chronic medical conditions such as Type-II diabetes, suggesting a role for fetal programming of endocrine dysfunction and metabolic regulation [2,3]. Consequently, parents who were expecting or delivered babies amidst pandemics may experience enduring impacts on their well-being, compounded by parenting children at elevated risk for stress-related changes in the early development and lifelong health of children [4,5]. Conceptual frameworks have been advanced regarding the lifelong effects of adversity in pregnancy and early childhood. As specified in the developmental origins of health hypothesis, parental stress interacts with environmental exposures (e.g., nutrition, pollution), to influence the maternal-fetal physiological feedback (as indicated by hormonal and inflammatory biomarkers) [6,7]. Parental behaviour and availability in the early postnatal period (e.g., parenting interaction and sensitivity) in turn shapes later biological, physical and neurodevelopmental outcomes [8]. In wake of the COVID-19 pandemic, toxic stress-informed frameworks for promotion of parental mental health during pregnancy have been expanded to include postnatal mental health and healthy parent-infant attachment amidst disrupted access to direct caregiving support [9]. Further, the ecobiodevelopmental framework illustrates how modifiable early environmental influences - such as unemployment, family poverty and access to healthcare - can impart an enduring effect on children's stress physiology and genetic expression [6,10,11]. An associated framework put forward by Nelson and Gabard-Durnam [12,13] suggests that we should view adversity as a violation of the expectable environment, with emphasis placed on the magnitude of this impact being greater during critical periods of brain development (such as the first 1000 days from conception to toddlerhood).

Emerging work is documenting the long-term implications of adversity related to the current pandemic including for example biological (i.e., COVID infection), acute environmental (i.e., temporary unemployment and psychosocial influences (i.e., impoverished, or atypical social environment) [14–16]. The social distancing restrictions and national lockdowns that were put in place to mitigate COVID-19 transmission have had a range of secondary consequences impacting the psychological wellbeing of pregnant women and new parents and the postnatal psychosocial environment that the infant is born into [17–19]. The shifts in socialization, stress and socio-economic position associated with COVID-19 public health guidance may have exacerbated the feelings of vulnerability, health vigilance and isolation associated with the adjustment to parenting. Heightened anxiety and depression were reported


For peer review only - http://bmjopen.bmj.com/site/about/guidelines.xhtml

during the national lockdown in the United Kingdom [20], with expectant and new mothers and fathers experiencing unique physical and psychological stressors [21,22] as well as constrained access to resources, especially with regard to family and caregiving support [23]. However, little is currently known about the impact of these COVID-related changes on the development of the infant. The current study aims to address this evidence gap by exploring the relationship regarding the family's reported experiences of these changes in terms of their impact on their pre and postnatal mental health, and interaction with their infant, and the potential subsequent impact of these changes on the infant's physical, sensory, affective, and cognitive development.

**The secondary impact of COVID-19 on pregnant women**

The COVID-19 pandemic has been the biggest public health emergency for over a century, necessitating extreme measures at a societal level to mitigate against death and prevent acute health services from being overwhelmed. These changes have led to a number of secondary consequences (i.e., increased caregiving demands for children and family members; isolation from family and community due to social distancing; job loss; financial hardship and increased interpersonal stressors or relationship violence), having a disproportionate and significant impact on women of childbearing age [23,24]. In the UK, the impact of the pandemic on this group of women has also been exacerbated by National Health Service (NHS) guidance that was produced in response to the national lockdown restrictions [25], in which hospital-based midwifery services placed limitations on partners being present during ultrasound visits and birth. In addition, most community-based services were discontinued, other than antenatal contact and new baby visits, all of which were required to be provided virtually unless otherwise indicated [26] with all other contacts being assessed and stratified according to vulnerability or clinical need (e.g., maternal mental health).

These changes not only affected the capacity of practitioners to support women during the perinatal period at a time of significantly heightened stress/distress [4] but also resulted in significant regional variations in access to healthcare and advice for expectant mothers across the UK. The changes have been associated with (i) a fourfold increase in stillbirths attributed to lack of preventive antenatal care, (ii) birthing partners denied access to the hospital for the birth or asked to leave immediately following the birth, and (iii) limited access to babies admitted to neonatal intensive care [27,28]. The NHS has also reported a reluctance on the part of parents to attend postnatal GP checks, due to parental attitudes related to COVID-19 infection (Institute of Health Visiting).

**The impact of COVID-19 on parental mental health during pregnancy**

Several online surveys conducted during the first national lockdown indicated that there was a significant increase in antenatal anxiety both in terms of pandemic-related pregnancy stress associated



For peer review only - http://bmjopen.bmj.com/site/about/guidelines.xhtml

with feeling unprepared for birth due to the pandemic, and stress related to fears of perinatal COVID-19 infection, with one large US survey (n = 4451) showing that around 30% of pregnant women experienced both types of stress [29]. Another US survey (n = 2740) that examined wider sources of stress showed that more than half of women reported increased stress in relation to concerns about food running out (59.2%, n = 1622), losing a job or household income (63.7%, n = 1745), or loss of childcare (56.3%, n = 1543). More than a third reported increased stress about conflict between household members (37.5%, n = 1028); and 93% (n = 2556) reported increased stress about getting infected with COVID-19 [30].

A number of online cross-sectional surveys also found significantly increased rates of anxiety and depression, based on the use of self-report standardised measures (e.g., Edinburgh Postnatal Depression Scale (EPDS); Hospital Anxiety and Depression Scale (HADS)). For example, a cross-sectional survey of 1,987 pregnant women in Canada in April 2020, found substantially elevated anxiety and depression symptoms, compared to similar pre-pandemic pregnancy cohorts; 37% reported clinically relevant symptoms of depression, and 57% reported clinically relevant symptoms of anxiety [31]. A second Canadian study found that a cohort of pregnant women, who were recruited during the COVID-19 pandemic, were twice as likely to present clinically significant levels of depressive and anxiety symptoms compared with a cohort of pregnant women recruited prior to the pandemic [32]. Early evidence in the UK similarly suggests that the impact on the mental health of pregnant women has been significant with heightened anxiety and depression being reported during the national lockdown (levels of mental distress rising from 18.9% (2018-19) to 27.3% in April 2020, one month into the national lockdown) [33].

This is of concern because there is consistent evidence to suggest that anxiety and depression in pregnancy can have a long-term impact on child development. For example, traumatic birth experiences amidst the changing public health situation or COVID-19 infection in the household have been associated with unusual parent-infant bonding [34]. Recent systematic reviews found that antenatal anxiety is associated with a range of adverse perinatal outcomes, including for example, premature delivery and low birthweight [35], in addition to a range of negative child outcomes that can persist into late adolescence, including an increased risk of child behaviour problems [36].

**The impact of COVID-19 on parental mental health, parent-infant interaction, and infant early environment**

There is growing evidence about how the pandemic and lockdown-related stressors impacted parental mental health during the postnatal period. Studies of postnatal depression suggest a similar picture to that prenatal, with around half of mothers caring for babies born during 2020 in the UK, reporting


For peer review only - http://bmjopen.bmj.com/site/about/guidelines.xhtml

feeling down, lonely and worried, with mental health symptoms exacerbated in mothers who travel to work, had a baby born prematurely or were from a lower-income household [37,38]. One Australian study that examined all online perinatal support forum posts related to COVID-19, from women between January 27 to May 12, 2020, showed that the content was predominantly negative, with around 63% being very or moderately negative. Negative words that were frequently used in the 831 posts included: "worried" (n=165, 19.9%), "risk" (n = 143, 17.2%), "anxiety" (n = 98, 11.8%), "concerns" (n = 74, 8.8%), and "stress" (n = 69, 8.3%) [38]. Similarly, first-time fathers who became parents during the onset of the pandemic in Italy reported greater stress than those with older children, and a study of fathers in Israel found that those who reported greater pandemic and parenting stress were more likely to report dysfunctional interactions with their infant and identify their baby's temperament as difficult [22,39].

Anxiety and depression in the postnatal period have been shown to affect the development of the infant because of the impact on the parent's interactions with their baby. For example, depressed mothers have been shown to be less sensitively attuned to infants, and less affirming and more negating of their experiences with their infant [40]. Babies of depressed mothers can exhibit deficits in their interpersonal functioning, such as less affective sharing, lower rates of interactive behaviour, poorer concentration, increased negative responses with strangers, and reduced secure attachment at 12 and 18 months [41,42]. Children of a mother who had postnatal depression are 42% more likely to experience depression by age 16 [43]. The prenatal and postnatal mental health of caregiving partners (including fathers) also appears to influence caregiver-infant interaction [44,45].

Although anecdotal reports of lockdown and work-from-home suggest more physical, social and material support for primary caregivers (from co-parents, fathers, non-marital partners, grandparents), and parents being more present and involved in caregiving for babies born during the pandemic compared to older siblings, the evidence to support this is currently lacking. Furthermore, it seems likely that any such benefits are socially determined: socioeconomically deprived families have unique vulnerability to the isolation of parenting amidst the pandemic [46,47]. Lower income families reported more frequent issues with breastfeeding, higher incidence of postnatal depression, and difficulty accessing caregiving and social support that might ameliorate the demands of caring for newborns [48–50].

In addition, limited exposure to infant peers or diverse social partners other than household members, or exposure to members of the public wearing masks, might confer different strategies for infant looking and communicative bids during social interactions. Whilst ongoing research is examining how the pandemic and its cascading effect on early contextual factors are affecting children ages 8-36 months,


For peer review only - http://bmjopen.bmj.com/site/about/guidelines.xhtml

there is a gap within the research observing earlier instances of development during the period referred to as the "baby blind-spot" (from pregnancy to 2 years old age) [18,47]. This study aims to bridge this gap by integrating pandemic-specific changes in parental mental health and COVID-19 induced social guidance as a unique context for infant development [51].

There also remains more questions than empirical answers at the present time regarding how 'stay at home' orders, lack of access to social support from family members and pandemic-specific stressors might have affected women at risk for domestic violence which was found to have increased significantly during the pandemic [21,52], and women heavily impacted by unemployment due to their overrepresentation in the retail, caregiving and hospitality workforce [52].

It is essential for longitudinal studies to address the impact of COVID-19 guidance and restrictions on the long-term development of the child [53], particularly in the UK as an example of a nation with an above-average COVID mortality rate, high-income disparity, and centralized healthcare system with a fairly uniform government response across regions with varying rates of infection. As such, our sample could serve as a test of the developmental programming hypothesis by assessing the extent to which a range of key domains of child development have been influenced by changes in stress and infant social exposure from pregnancy to early infancy, arising from the social restrictions in place at different points during the pandemic. This study has two main goals: (i) to examine how parent's experiences of the social, medical, and financial changes during the pandemic have impacted pre and postnatal parental mental health and parent-infant social interaction; and (ii) to investigate the extent to which these COVID related changes in parental pre and postnatal mental health and social interaction are associated with fetal and infant development.

## STUDY DESIGN

The COVID in the Context of Pregnancy, Infancy and Parenting (CoCoPIP) study, is a national online survey being carried out in the UK, which was widely advertised from July 2020, that continues to actively recruit families for participation. The research comprises a mixed-methods study collecting data inclusive of both; a) validated physical and psychological assessments, and b) open-ended questions to allow the participant to elaborate on their experience in their own words. The large sample collected enables us to use data-driven (lasso, Bayesian with infants born pre-pandemic as dictating priors) and hypothesis-driven approaches to assess if COVID-19 provides a model for how individual factors (maternal mental health, birth timing, caregiving) interact with institutional factors (government lockdowns, social and financial support, etc.).


For peer review only - http://bmjopen.bmj.com/site/about/guidelines.xhtml

The CoCoPIP Study addresses four key hypotheses (H1-4) (see *Figure 1*). Our variable selection and sequential building of hypotheses embed our key frameworks: i) examining parental mental health in light of stress and social, financial and contextual factors (ecobiodevelopmental model); ii) how infant sensory processing pertains to caregiving and social exposure of infants, relative to lockdown/COVID-19 transmission during infant's birth, family COVID-19 vigilance and parenting anxiety (early expectable environment); iii) the interaction of maternal mental health and fetal growth measures as longitudinal predictors of infant cognitive outcomes (developmental origins of disease hypothesis); iv) finally, encompassing the social, financial and contextual factors which impact parental mental health to shape infant temperament and sensory processing, accounting for early infant caregiving and social environment (developmental programming). Ultimately, our research programme can demonstrate support (or lack thereof) for extending developmental programming-based frameworks beyond child physical health (insulin resistance, stature, etc.) [54,55] and cognitive outcomes [56–58], to explain variability in proximal domains such as infant affective, social and sensory capacities.

**[Insert figure 1]**

**Eligibility criteria and recruitment strategies**

Eligibility criteria for the study is expectant parents (at any stage of pregnancy) or parents of an infant between the ages of 0-6 months. Either parent can take part, with questionnaires being adapted to the parents' status as mother or father. The study is open to parents who had a baby within 6 months prior to the first period of lockdown in the UK (23rd March 2020) as well as continuing to collect information from parents during the current and future changes in COVID-related health and societal restrictions.

For optimal national representation across the UK, recruitment strategies include (i) targeting NHS antenatal classes and National Childbirth Trust (NCT) groups identified nationally, with an emphasis on areas of low socio-economic status (SES) using the government indices of multiple deprivation (IMD) and rural areas without access to NCT groups, (ii) partnering with NHS/National Institute for Health Research (NIHR) collaborative sites and charity and policy group partners (e.g., The Brazelton Centre UK, Centre for Health and the Public Interest) to widen knowledge of the survey, (iii) posting online via social media platforms (e.g., Twitter) and public sharing to facilitate snowball sampling and (iv) targeting populations experiencing increased local lockdown measures as, and when, COVID-19 rates and related policy change across the UK. Whilst recruitment efforts have been focused on the UK, the survey is currently open to all expectant and new families worldwide. Participation in the survey is incentivized using the offer of a chance to win a £100 digital gift card (on receipt participants are able to select from either an Amazon® or one4all® gift card). A prize is drawn for every 100 participants who complete the survey, giving a 1/100 chance of winning at each time point that they complete.


For peer review only - http://bmjopen.bmj.com/site/about/guidelines.xhtml

**Patient and Public Involvement**

The study was designed with input from the public, particularly pregnant and new parents who had an infant during the onset of the pandemic from March – May 2020. Input included aspects such as wording of questions and ease of completing questionnaires (both visually and in length). Results will be disseminated to study participants through various social media platforms that participants are given links to during initial recruitment and in subsequent follow-up correspondence.

**Power Calculation**

To ensure sufficient power for the study, statistical power calculations (G*Power adapted for regression) based on three outcomes, up to three predictors and four co-variables, estimated a minimum sample size for Hypothesis 1 of n = 400 (small effect, $f^2 = 0.02$). Statistical power calculations were based on a study of sociodemographic control variables, traumatic event impact scale and pregnancy-specific anxiety [37]. In the same manner, for Hypothesis 2, a minimum sample size of n = 800 (small effect, n = 400 infants x 2 postnatal timepoints) is required, based on ongoing analyses by our group on parent-infant social interaction data [38]. For Hypothesis 3 and 4, we will minimise data loss using post-hoc assignment of families to an accelerated longitudinal design - this requires a minimum sample size of n = 500 (small effect) using a study on acute disasters, parental mental health and infant development [39]. With timing and cross lag accounted for, and attrition rate of 30% - assuming current pattern of 80% of parents' consenting to be contacted again (as indicated by our pilot survey) – a minimum cohort sample of n = 1500 is required.

**Study Measures**

The online survey is logic-dependent and adaptive, only showing questions relevant to the parent's current situation (e.g., antenatal or with an infant of 2 months of age) in relation to the following six time points: the second and third trimester of pregnancy; infant aged 0-3 months, infant aged 3-6 months; and toddler aged 12- and 18-months. The following data is collected: (i) parental mental health and attitudes, (ii) healthcare access and support during pregnancy and birth, (iii), fetal physical development and infant social and cognitive development, (iv) direct impact of COVID-19 on daily lives and lastly (v) developmental outcomes in infants born during the pandemic. *Table 1* provides an overview of the measures used timepoints (for a detailed summary of the measures and questionnaires used within the survey see *suppl file 1 and suppl Table 1)*.

**Follow-ups and reminders**

Participants are invited to take part in a follow-up survey at the end of the initial survey. Where they consent to this, they are contacted via email containing a link to the separate online survey. The follow-



For peer review only - http://bmjopen.bmj.com/site/about/guidelines.xhtml

up survey has been condensed to include follow-up questionnaires only (*see Table 1* and *Figure 2* for participant follow-up flowchart). The appropriate time for follow-up is calculated based on the ages (infant or fetal gestation) provided by the participant at initial recruitment (see *Figure 2* for project timeline).

## DATA ANALYSIS PLAN

The Statistical Analysis Plan was developed based upon the UK Dept of Health/Medical Research Council Clinical Trials Toolkit and NHS epidemiological study designs, with details outlined per the standards for random control trials and clinical trials [59,60].

### Quality Control

Ongoing quality control is evaluated bi-weekly. All data is checked for accuracy and invalid data is removed. Study data is collected and managed using Research Electronic Data Capture (REDCap®) tools hosted at the University of Cambridge [61]. REDCap® is a secure, web-based software platform designed to support data capture for research studies, providing 1) an intuitive interface for validated data capture; 2) audit trails for tracking data manipulation and export procedures; 3) automated export procedures for seamless data downloads to common statistical packages; and 4) procedures for data integration and interoperability with external sources. Personal data (e.g., caregiver DOB, email address) is stored securely within a password encrypted electronic databased isolated from the research data. Access to the data is fully audited to ensure data security is governed by a management team and in compliance with ethical guidelines [62].

For peer review only

| | Timepoint | | | | | |
|---|---|---|---|---|---|---|
| | Pregnancy | | Infant | | Toddler | |
| | 1 | 2 | 3 | 4 | 5 | 6 |
| **Section A: Consent and participant background information** | | | | | | |
| Consent[a] | x | x | x | x | | |
| Demographics[b] | x | x | x | x | | |
| Income and employment status | x | x | x | x | | |
| **Section B: Pregnancy measures** | | | | | | |
| Fetal growth measures and pregnancy | x[c] | x[d] | | | | |
| Health care support and access | x | x | x | x | | |
| Antenatal Emotional Attachment Scale (AEAS) | x | x | | | | |
| Pregnancy Related Anxiety Questionnaire Revised (PRAQ-R) | x | x | | | | |
| **Section C: Infant birth and development measures** | | | | | | |
| Birth information[e] | | | x | x | | |
| Infant Behaviour Questionnaire (IBQ) | | | | x | x | |
| Infant Toddler Sensory Profile (ITSP) | | | x | x | x | |
| Infant related anxiety | | | x | x | | |
| Face-to-face interaction index | | | x | x | | |
| **Section D: Toddler Development measures** | | | | | | |
| Ages and Stages | | | | | x | |
| Oxford CDI | | | | | x | |
| Q-CHAT | | | | | | x |
| Vineland parent and caregiver form | | | | | | x |
| **Section E: Parental mental health and support measures** | | | | | | |
| State Trait Anxiety Index – State (STAI-S) | x | x | x | x | x | x |
| Caregiving, social interaction and support questionnaire | | | x | x | | |
| Stressful life events questionnaire | | | | | x | x |
| **Section F: Parenting and family measures** | | | | | | |
| Parenting reflective functioning questionnaire (PRFQ) | | | | | x | |
| Comprehensive early childhood parenting questionnaire (CECPAQ) | | | | | x | |
| **Section G: COVID Impact** | | | | | | |
| COVID situational influences | x | x | x | x | | |
| COVID health report | x | x | x | x | | |
| COVID concern and event impact scale | x | x | x | x | | |
| Social distance impacts | x | x | x | x | | |
| Vaccine | x | x | x | x | | |
| Difficulties in Emotion Regulation Scale (DERS) | | | | | x | |

[a-b] Participants will only be asked to complete this section once, when they initially join the study. The study can be joined at any timepoint. Those eligible will be asked if they wish to participate longitudinally.

[c] only physical questions in relation to 2nd trimester scan.

[d] only physical questions in relation to 3rd trimester scan.

[e] will only be asked to complete this section once

*Table 1: A summary of assessments and questionnaires used separated by time point.*

For peer review only



For peer review only - http://bmjopen.bmj.com/site/about/guidelines.xhtml

**Analysis plan**

Overall, we aim to identify which stress-associated moderators (i.e., loss of income, COVID-19 illness, local access to ante/postnatal support) impact significantly on parental mental health, and in turn, infant development. Further to the plans outlined for each aim below, tests of normality and sensitivity analyses (comparing observed values and imputed missing values) will be conducted. Non-linear tests of significance and interpolation approaches will be applied where appropriate.

To address Hypothesis 1 (*see Figure 1*), a combination of quantitative and qualitative analyses will be undertaken. Structural equation modelling (SEM) and hypothesis-driven regressions will explore how multiple aspects of pre- and post-natal family support (social, financial and health) are associated with latent outcomes of stress (parenting anxiety and pandemic-related stress) and latent outcomes of mental health symptoms. An inductive approach to data analysis will be undertaken [63] for the open-ended qualitative data, and we will use NVivo (QSR International Pty Ltd) to code the data. Following this, several approaches including thematic [63], sentiment and context content analysis will be undertaken using a natural language processing (NLP) approach [64] (machine learning) to identify forms of social, medical and financial support in relation to the valence of parental attitudes. Regression analyses will then be conducted to understand the directional relationship between resulting latent factors (qualitative responses and quantitative data) and parental mental health.

To address Hypothesis 2, Bayesian non-linear regressions will be used to explore how COVID-19 related restrictions during an infant's birth altered infant social exposure with caregivers and non-household social partners, and the impact of these in turn on infant processing of sounds, sights and social stimuli. The dependent variables will be derived from scores of the infant toddler sensory profile, a standard assessment of infant self-regulation and responsiveness to their environment. On the first level, the timing of an infant's birth will be coded based on whether a lockdown or no lockdown was imposed by the government, as well as coding for more specific shifts in the UK government public health policy and 'unlocking' guidance from July 14 2020 to July 19 2021. Additional COVID-19 factors may be entered, including suspected or positive cases in loved ones, parental COVID-19 concern, pandemic-related parenting anxiety and parent-reported adherence to lockdown. On the next level, infant's social exposure will draw from caregiver-reported of their frequency of face-to-face interactions with their baby, as well as their baby's exposure to social partners from outside the household, in-person, at a distance and online. On the third level, family sociodemographic factors, such as the number of family members in each household, family income, ethnicity and high-risk health conditions will be included.



For peer review only - http://bmjopen.bmj.com/site/about/guidelines.xhtml

For peer review only

To address Hypothesis 3, linear regressions will be used to explore the influence of maternal mental health longitudinally on the developing offspring across pre- to post-natal life: from fetal (12-weeks/20 weeks gestational age) to 18 months of age. After testing for normality and transforming variables accordingly, standardised z-scores will be created from the collected fetal growth measurements (i.e., head circumference, femur length and abdominal circumference), which will then be transformed into a composite score accounting for gestational age and/or estimated fetal weight at time of scan to be used for analysis. Z-scores will also be computed and used where appropriate within the analysis (e.g., infant-toddler sensory profile).

To address Hypothesis 4, outputs from Hypothesis 1 and 2 (impact of COVID-19 on parental mental health and infant social interactions) will be nominated using lasso regression coefficients in relation to longitudinal social and cognitive child development domains (assessing language, motor sensory and the early emergence of developmental conditions) of the infant/toddler across the 0-18 months of life using SEM, applying full information maximum likelihood to account for missingness and to identify developmental-hypothesis driven clusters of affected families by factors such as birth timing, individual family stressors and pandemic restrictions during survey.

## CURRENT COHORT DESCRIPTION AND DEMOGRAPHICS

Initiated in July 2020, this study is ongoing with n = 1700 families currently enrolled (6$^{th}$ May 2021). Parents can consent to complete the questionnaire up to six times during pregnancy/parenting until their infants are 18 months of age. For those participants who contribute more than one time point (between antenatal and postnatal timepoints ≤ 6 months) an invitation is issued for a follow up to assess their toddler's development when aged 12 and 18 months (see *Figure 2* for study flow chart).

To date 1700 of families have participated in at least one time-point of the study, with 641 families joining at time points 1-2, 372 families at time-point 3, and 687 families at time-point 4 (see *Figure 2 and Table 1* for time-points). 61% of these families have consented to completing the subsequent follow-up sections of the study. To date 97.4% of respondents, identify as mothers, 2.3% as fathers and 0.3% as another parent or caregiver, with the majority of participating families disclosing their ethnicity as white (89.2%). Those participating families who are from the UK have their household information (i.e., household income, location of participating families, index of multiple deprivation and respondent's education level) described in *Figure 3*.

**[Insert figure 3]**



For peer review only - http://bmjopen.bmj.com/site/about/guidelines.xhtml

# ETHICS AND DISSEMINATION

Ethics approval for the survey was given by the University of Cambridge, Psychology Research Ethics Committee (PREC) (PRE.2020.077). All respondents are required to be over the age of 18 years and give electronic informed consent. Caregivers agreeing to be followed-up longitudinally give consent at each timepoint and are made aware that their participation can be stopped at any time within the study. Permissions have been obtained from participants to ensure anonymised data can be made available on open-source platforms.

A proactive dissemination pathway has been established from the outset. We will engage with policy stakeholders (health practitioners/Department of Health) and social media platforms to create discussion around this topic. Dissemination of findings will be via public forums (i.e., social media, media, collaborators family dissemination pathways) and at the national (i.e., NHS England/NHS Improvement, Royal College of Paediatrics and Child Health, Centre for Health and the Public Interest) and local (Cambridge Universities Hospitals NHS Foundation Trust) level. Data will continue to be disseminated throughout the period of the study to promote discussion and raise the profile of the population identified as being one of the most vulnerable and neglected during the pandemic.

To date, qualitative responses from the first five months of data collection have been analysed to explore parents experiences of being pregnant in relation to healthcare access during the pandemic [19]. This was conducted using thematic and sentiment analysis. The initial findings suggest that a range of adverse effects have been experienced by expectant parents in the UK relating to changes in antenatal support and healthcare appointments in response to governmental guidance with regard to social distancing. These findings point to an urgent need to better address the unique health care needs of each pregnant woman going forward.

## Data Sharing Plan

Questionnaires and study goals were made available upon request using the Open Science Foundation platform in July 2020 and made public at https://osf.io/m7zuw/ in August 2020. Study protocol, follow-up questionnaires and statistical analysis code will be uploaded and shared to facilitate data sharing and collaboration, in accordance with Research, Innovation and Science Policy Experts (RISE) EU principles [65]. Qualitative data generated and analysed during the study will not be made publicly available due to ethical and privacy restrictions, however researchers can submit a research proposal to the Data Sharing Management Committee to request access and collaboration.


For peer review only - http://bmjopen.bmj.com/site/about/guidelines.xhtml

**Acknowledgements:** We are extremely grateful to all those families who gave their time to participate and to Esther Adememo, Maddie Walton and Zahra Khan who worked on the CoCoPIP study during their undergraduate and master's studies at the University of Cambridge.

**Author statement: Ezra Aydin:** Conceptualization, Methodology, Writing - Original Draft. **Staci M. Weiss:** Conceptualization, Methodology, Writing - Original Draft. **Kevin A. Glasgow:** Methodology, Visualisation, Writing - Review & Editing **Topun Austin:** Methodology, Supervision, Writing - Review & Editing. **Mark H. Johnson:** Supervision, Funding acquisition, Writing - Review & Editing. **Jane Barlow:** Supervision, Writing - Review & Editing. **Sarah Lloyd-Fox:** Conceptualization, Methodology, Supervision, Funding acquisition, Writing - Review & Editing.

**Funding source:** This research was funded by a Medical Research Council Programme Grant MR/T003057/1 to MJ, and an UKRI Future Leaders fellowship (MRC grant MR/S018425/1) to SLF. The views expressed are those of the authors and not necessarily those of the MRC or the UKRI.

The NIHR Cambridge Biomedical Research Centre (BRC) is a partnership between Cambridge University Hospitals NHS Foundation Trust and the University of Cambridge, funded by the National Institute for Health Research (NIHR), T.A. is supported by the NIHR Cambridge Biomedical Research Centre (BRC). TA is also supported by the NIHR Brain Injury MedTech Co-operative. The views expressed are those of the author(s) and not necessarily those of the NIHR or the Department of Health and Social Care.

**Disclosure Statement:** The authors have no potential conflicts of interest to disclose.

For peer review only

**Figure Legends**

*Figure 1: Covid in the Context of Pregnancy, Infancy and Parenting (CoCoPIP) study four key hypotheses.*

A participant can join the study at any of the above 3 underlined timepoints

*Participants are only followed up at these two timepoints if they have participated in at least two previous timepoints.

** Projected participant follow-up completion dates.

*Figure 2: Flow diagram of COVID in the Context of Pregnancy, Infancy and Parenting (CoCoPIP) study follow up participation.*

*Figure 3: Bubble map depicting spread of participations location in the UK (if postcode was provided) with respondent's education level, Index of Multiple Deprivation (IMD) and household income breakdown reported on the right.*

For peer review only

## REFERENCES


1 Stein AD, Ravelli AC, Lumey LH. Famine, third-trimester pregnancy weight gain, and intrauterine growth: the Dutch Famine Birth Cohort Study. *Hum Biol* 1995;**67**:135–50.

2 Mink J, Boutron-Ruault M-C, Charles M-A, *et al.* Associations between early-life food deprivation during World War II and risk of hypertension and type 2 diabetes at adulthood. *Sci Rep* 2020;**10**:5741. doi:10.1038/s41598-020-62576-w

3 Laplante DP, Brunet A, Schmitz N, *et al.* Project Ice Storm: Prenatal Maternal Stress Affects Cognitive and Linguistic Functioning in 5½-Year-Old Children. *J Am Acad Child Adolesc Psychiatry* 2008;**47**:1063–72. doi:10.1097/CHI.0b013e31817eec80

4 Guo J, De Carli P, Lodder P, *et al.* Maternal mental health during the COVID-19 lockdown in China, Italy, and the Netherlands: a cross-validation study. *Psychol Med* 2021;:1–11. doi:10.1017/S0033291720005504

5 Tucci V, Moukaddam N, Meadows J, *et al.* The forgotten plague: Psychiatric manifestations of ebola, zika, and emerging infectious diseases. *J Glob Infect Dis* 2017;**9**:151. doi:10.4103/jgid.jgid_66_17

6 King S, Dancause K, Turcotte-Tremblay A-M, *et al.* Using Natural Disasters to Study the Effects of Prenatal Maternal Stress on Child Health and Development: Natural Disasters and Prenatal Maternal Stress. *Birth Defects Res Part C Embryo Today Rev* 2012;**96**:273–88. doi:10.1002/bdrc.21026

7 Gluckman PD, Hanson MA, Buklijas T. A conceptual framework for the developmental origins of health and disease. *J Dev Orig Health Dis* 2010;**1**:6–18. doi:10.1017/S2040174409990171

8 Wadhwa P, Buss C, Entringer S, *et al.* Developmental Origins of Health and Disease: Brief History of the Approach and Current Focus on Epigenetic Mechanisms. *Semin Reprod Med* 2009;**27**:358–68. doi:10.1055/s-0029-1237424

9 Iqbal A, Burrin C, Aydin E, *et al.* Generation COVID-19 – Should the foetus be worried? *Acta Paediatr* 2020;:apa.15693. doi:10.1111/apa.15693

10 Nelson III CA, Gabard-Durnam LJ. Early adversity and critical periods: neurodevelopmental consequences of violating the expectable environment. *Trends Neurosci* 2020;**43**:133–43.

11 Humphreys KL, Salo VC. Expectable environments in early life. *Curr Opin Behav Sci* 2020;**36**:115–9. doi:10.1016/j.cobeha.2020.09.004

12 Shonkoff JP, Garner AS, THE COMMITTEE ON PSYCHOSOCIAL ASPECTS OF CHILD AND FAMILY HEALTH, COMMITTEE ON EARLY CHILDHOOD, ADOPTION, AND DEPENDENT CARE, AND SECTION ON DEVELOPMENTAL AND BEHAVIORAL PEDIATRICS, *et al.* The Lifelong Effects of Early Childhood Adversity and Toxic Stress. *Pediatrics* 2012;**129**:e232–46. doi:10.1542/peds.2011-2663



For peer review only - http://bmjopen.bmj.com/site/about/guidelines.xhtml

13 Folger AT, Nidey N, Ding L, *et al.* Association between maternal adverse childhood experiences and neonatal SCG5 DNA methylation—effect modification by prenatal home visiting. *Am J Epidemiol* 2021;:kwab270. doi:10.1093/aje/kwab270

14 Davies C, Hendry A, Gibson SP, *et al.* Early childhood education and care ( ECEC ) during COVID -19 boosts growth in language and executive function. *Infant Child Dev* 2021;**30**. doi:10.1002/icd.2241

15 Guo P, Ballesteros AB, Yeung SP, *et al.* COVCOG 1: Factors predicting Cognitive Symptoms in Long COVID. A First Publication from the COVID and Cognition Study. Epidemiology 2021. doi:10.1101/2021.10.26.21265525

16 Lawson M, Piel MH, Simon M. Child Maltreatment during the COVID-19 Pandemic: Consequences of Parental Job Loss on Psychological and Physical Abuse Towards Children. *Child Abuse Negl* 2020;**110**:104709. doi:10.1016/j.chiabu.2020.104709

17 Ye J. Pediatric Mental and Behavioral Health in the Period of Quarantine and Social Distancing With COVID-19. *JMIR Pediatr Parent* 2020;**3**:e19867. doi:10.2196/19867

18 Martin-Key NA, Spadaro B, Schei TS, *et al.* Proof-of-Concept Support for the Development and Implementation of a Digital Assessment for Perinatal Mental Health: Mixed Methods Study. *J Med Internet Res* 2021;**23**:e27132. doi:10.2196/27132

19 Aydin E, Glasgow KA, Weiss SM, *et al.* Expectant parents' perceptions of healthcare and support during COVID-19 in the UK: a thematic analysis. *J Reprod Infant Psychol* 2022;:1–13. doi:10.1080/02646838.2022.2075542

20 Davenport MH, Meyer S, Meah VL, *et al.* Moms are not ok: COVID-19 and maternal mental health. *Front Glob Womens Health* 2020;**1**:1.

21 Barlow J, Bach-Mortensen A, Homonchuk O, *et al.* The impact of the COVID-19 pandemic on services from pregnancy through age 5 years for families who are high risk of poor outcomes or who have complex social needs. 2020.

22 Ben-Yaakov O, Ben-Ari OT. COVID-19-Related anxieties and parenting stress among first-time mothers and fathers in their first year of parenthood. *Psychol Health* 2021;:1–15. doi:10.1080/08870446.2021.1942875

23 Di Giorgio E, Di Riso D, Mioni G, *et al.* The interplay between mothers' and children behavioral and psychological factors during COVID-19: an Italian study. *Eur Child Adolesc Psychiatry* 2021;**30**:1401–12. doi:10.1007/s00787-020-01631-3

24 Shah SMA, Mohammad D, Qureshi MFH, *et al.* Prevalence, Psychological Responses and Associated Correlates of Depression, Anxiety and Stress in a Global Population, During the Coronavirus Disease (COVID-19) Pandemic. *Community Ment Health J* 2021;**57**:101–10. doi:10.1007/s10597-020-00728-y

25 Davis N. NHS trusts begin suspending home births due to coronavirus. 2020.

26 NHS. Supporting Pregnant women using maternity service during the coronavirus pandemic: Actions for NHS providers. 2020.


For peer review only - http://bmjopen.bmj.com/site/about/guidelines.xhtml

27 Sherwood H. Midwife shortage doubles as NHS staff diverted to tend Covid-19 patients. 2020.

28 Khalil A, Von Dadelszen P, Draycott T, *et al.* Change in the incidence of stillbirth and preterm delivery during the COVID-19 pandemic. *Jama* 2020;**324**:705–6.

29 Preis H, Mahaffey B, Heiselman C, *et al.* Vulnerability and resilience to pandemic-related stress among U.S. women pregnant at the start of the COVID-19 pandemic. *Soc Sci Med* 2020;**266**:113348. doi:10.1016/j.socscimed.2020.113348

30 Moyer CA, Compton SD, Kaselitz E, *et al.* Pregnancy-related anxiety during COVID-19: a nationwide survey of 2740 pregnant women. *Arch Womens Ment Health* 2020;**23**:757–65. doi:10.1007/s00737-020-01073-5

31 Lebel C, MacKinnon A, Bagshawe M, *et al.* Elevated depression and anxiety symptoms among pregnant individuals during the COVID-19 pandemic. *J Affect Disord* 2020;**277**:5–13. doi:10.1016/j.jad.2020.07.126

32 Berthelot N, Lemieux R, Garon-Bissonnette J, *et al.* Uptrend in distress and psychiatric symptomatology in pregnant women during the coronavirus disease 2019 pandemic. *Acta Obstet Gynecol Scand* 2020;**99**:848–55. doi:10.1111/aogs.13925

33 Pierce M, Hope H, Ford T, *et al.* Mental health before and during the COVID-19 pandemic: a longitudinal probability sample survey of the UK population. *Lancet Psychiatry* 2020;**7**:883–92. doi:10.1016/S2215-0366(20)30308-4

34 Grigoriadis S, Graves L, Peer M, *et al.* Maternal Anxiety During Pregnancy and the Association With Adverse Perinatal Outcomes: Systematic Review and Meta-Analysis. *J Clin Psychiatry* 2018;**79**. doi:10.4088/JCP.17r12011

35 Stein A, Pearson RM, Goodman SH, *et al.* Effects of perinatal mental disorders on the fetus and child. *The Lancet* 2014;**384**:1800–19. doi:10.1016/S0140-6736(14)61277-0

36 Dib S, Rougeaux E, Vázquez-Vázquez A, *et al.* Maternal mental health and coping during the COVID-19 lockdown in the UK: Data from the COVID-19 New Mum Study. *Int J Gynecol Obstet* 2020;**151**:407–14. doi:10.1002/ijgo.13397

37 Ahmad M, Vismara L. The Psychological Impact of COVID-19 Pandemic on Women's Mental Health during Pregnancy: A Rapid Evidence Review. *Int J Environ Res Public Health* 2021;**18**:7112. doi:10.3390/ijerph18137112

38 Chivers BR, Garad RM, Boyle JA, *et al.* Perinatal Distress During COVID-19: Thematic Analysis of an Online Parenting Forum. *J Med Internet Res* 2020;**22**:e22002. doi:10.2196/22002

39 Mangiavacchi L, Piccoli L, Pieroni L. Fathers matter: Intrahousehold responsibilities and children's wellbeing during the COVID-19 lockdown in Italy. *Econ Hum Biol* 2021;**42**:101016. doi:10.1016/j.ehb.2021.101016

40 Murray L, Fiori-Cowley A, Hooper R, *et al.* The Impact of Postnatal Depression and Associated Adversity on Early Mother-Infant Interactions and Later Infant Outcome. *Child Dev* 1996;**67**:2512. doi:10.2307/1131637



For peer review only - http://bmjopen.bmj.com/site/about/guidelines.xhtml

41 Murray L. The Impact of Postnatal Depression on Infant Development. *J Child Psychol Psychiatry* 1992;**33**:543–61. doi:10.1111/j.1469-7610.1992.tb00890.x

42 Stein A, Gath DH, Bucher J, *et al.* The Relationship between Post-natal Depression and Mother–Child Interaction. *Br J Psychiatry* 1991;**158**:46–52. doi:10.1192/bjp.158.1.46

43 Murray L, Arteche A, Fearon P, *et al.* Maternal Postnatal Depression and the Development of Depression in Offspring Up to 16 Years of Age. *J Am Acad Child Adolesc Psychiatry* 2011;**50**:460–70. doi:10.1016/j.jaac.2011.02.001

44 Amodia-Bidakowska A, Laverty C, Ramchandani PG. Father-child play: A systematic review of its frequency, characteristics and potential impact on children's development. *Dev Rev* 2020;**57**:100924. doi:10.1016/j.dr.2020.100924

45 Foley S, Hughes C. Great expectations? Do mothers' and fathers' prenatal thoughts and feelings about the infant predict parent-infant interaction quality? A meta-analytic review. *Dev Rev* 2018;**48**:40–54. doi:10.1016/j.dr.2018.03.007

46 Mayopoulos GA, Ein-Dor T, Dishy GA, *et al.* COVID-19 is associated with traumatic childbirth and subsequent mother-infant bonding problems. *J Affect Disord* 2021;**282**:122–5. doi:10.1016/j.jad.2020.12.101

47 Brown A, Shenker N. Experiences of breastfeeding during COVID-19: Lessons for future practical and emotional support. *Matern Child Nutr* 2021;**17**. doi:10.1111/mcn.13088

48 Barlow J, Underdown A. Child maltreatment during infancy: atypical parent–infant relationships. *Paediatr Child Health* 2018;**28**:114–9. doi:10.1016/j.paed.2017.11.001

49 Petterson SM, Albers AB. Effects of Poverty and Maternal Depression on Early Child Development. *Child Dev* 2001;**72**:1794–813. doi:10.1111/1467-8624.00379

50 Pearson RM, Evans J, Kounali D, *et al.* Maternal Depression During Pregnancy and the Postnatal Period: Risks and Possible Mechanisms for Offspring Depression at Age 18 Years. *JAMA Psychiatry* 2013;**70**:1312. doi:10.1001/jamapsychiatry.2013.2163

51 European Parliament. Directorate General for Internal Policies of the Union. *The gendered impact of the Covid-19 crisis and post-crisis period.* LU: : Publications Office 2020. https://data.europa.eu/doi/10.2861/393602 (accessed 15 Mar 2021).

52 Cook R, Grimshaw D. A gendered lens on COVID-19 employment and social policies in Europe. *Eur Soc* 2021;**23**:S215–27. doi:10.1080/14616696.2020.1822538

53 Fiske A, Scerif G, Holmboe K. Maternal depressive symptoms and early childhood temperament before and during the COVID-19 pandemic in the United Kingdom. PsyArXiv 2021. doi:10.31234/osf.io/quket

54 Buss C, Davis EP, Muftuler LT, *et al.* High pregnancy anxiety during mid-gestation is associated with decreased gray matter density in 6-9-year-old children. *Psychoneuroendocrinology* 2010;**35**:141–53. doi:10.1016/j.psyneuen.2009.07.010

55 Scheinost D, Sinha R, Cross SN, *et al.* Does prenatal stress alter the developing connectome? *Pediatr Res* 2017;**81**:214–26. doi:10.1038/pr.2016.197



For peer review only - http://bmjopen.bmj.com/site/about/guidelines.xhtml

56 Buss C, Davis EP, Hobel CJ, *et al.* Maternal pregnancy-specific anxiety is associated with child executive function at 6–9 years age. *Stress* 2011;**14**:665–76. doi:10.3109/10253890.2011.623250

57 Thomas JC, Letourneau N, Campbell TS, *et al.* Developmental origins of infant emotion regulation: Mediation by temperamental negativity and moderation by maternal sensitivity. *Dev Psychol* 2017;**53**:611–28. doi:10.1037/dev0000279

58 Letcher P, Greenwood CJ, Romaniuk H, *et al.* Adolescent and young adult mental health problems and infant offspring behavior: Findings from a prospective intergenerational cohort study. *J Affect Disord* 2020;**272**:521–8. doi:10.1016/j.jad.2020.03.101

59 Gamble C, Krishan A, Stocken D, *et al.* Guidelines for the Content of Statistical Analysis Plans in Clinical Trials. *JAMA* 2017;**318**:2337. doi:10.1001/jama.2017.18556

60 Hemming K, Kearney A, Gamble C, *et al.* Prospective reporting of statistical analysis plans for randomised controlled trials. *Trials* 2020;**21**:898, s13063-020-04828–8. doi:10.1186/s13063-020-04828-8

61 Harris PA, Taylor R, Thielke R, *et al.* Research electronic data capture (REDCap)—A metadata-driven methodology and workflow process for providing translational research informatics support. *J Biomed Inform* 2009;**42**:377–81. doi:10.1016/j.jbi.2008.08.010

62 Staniszewska S, Brett J, Simera I, *et al.* GRIPP2 reporting checklists: tools to improve reporting of patient and public involvement in research. *BMJ* 2017;:j3453. doi:10.1136/bmj.j3453

63 Pope C, Ziebland S, Mays N. Qualitative research in health care. Analysing qualitative data. *BMJ* 2000;**320**:114–6. doi:10.1136/bmj.320.7227.114

64 Leeson W, Resnick A, Alexander D, *et al.* Natural Language Processing (NLP) in Qualitative Public Health Research: A Proof of Concept Study. *Int J Qual Methods* 2019;**18**:160940691988702. doi:10.1177/1609406919887021

65 European Commission. Directorate General for Research and Innovation. *Europe's future: open innovation, open science, open to the world : reflections of the Research, Innovation and Science Policy Experts (RISE) High Level Group.* LU: : Publications Office 2017. https://data.europa.eu/doi/10.2777/348700 (accessed 20 May 2021).



For peer review only - http://bmjopen.bmj.com/site/about/guidelines.xhtml

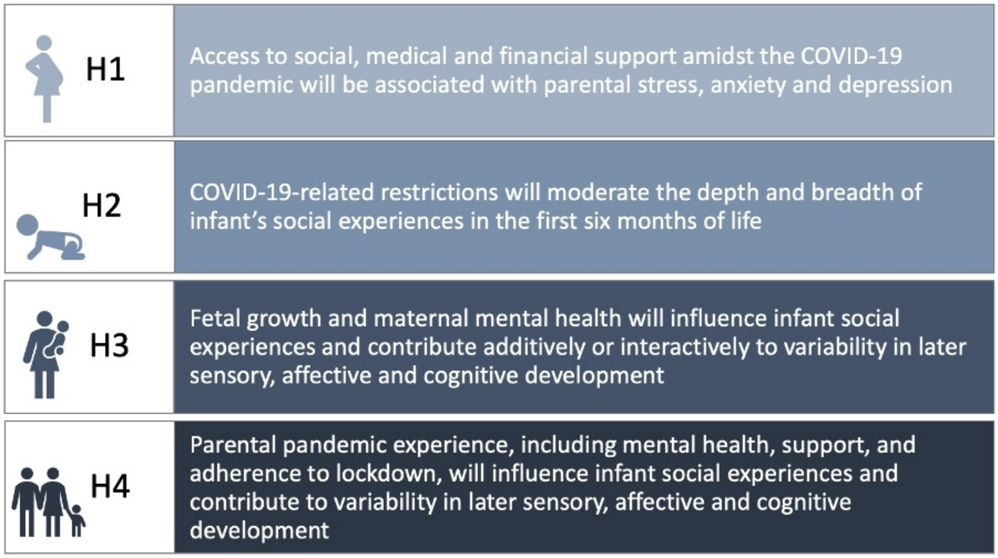


Figure 1: Covid in the Context of Pregnancy, Infancy and Parenting (CoCoPIP) study four key hypotheses.

159x89mm (144 x 144 DPI)

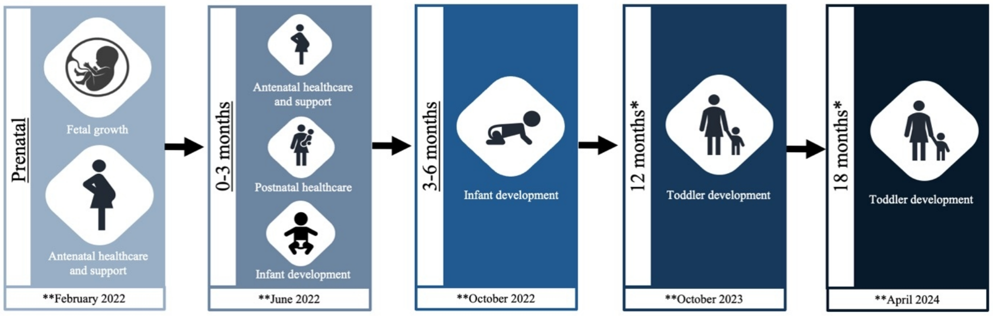


A participant can join the study at any of the above 3 underlined timepoints
*Participants are only followed up at these two timepoints if they have participated in at least two previous timepoints.
** Projected participant follow-up completion dates.
Figure 2: Flow diagram of Covid in the Context of Pregnancy, Infancy and Parenting (CoCoPIP) study follow up participation.

159x51mm (220 x 220 DPI)

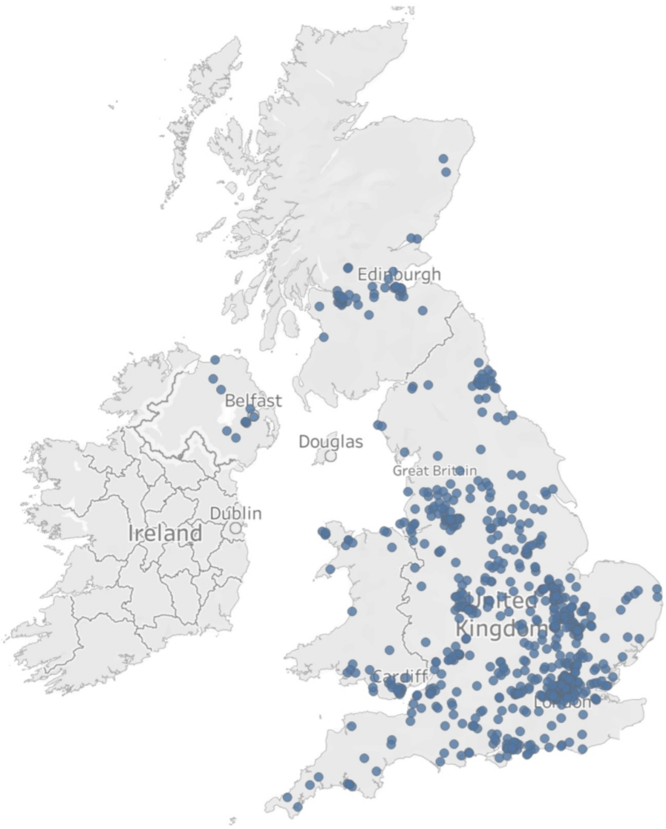


| Respondents Education Level | |
|---|---|
| | n (%) |
| School ≤ 16 years old | 48 (3.2) |
| College ≤ 18 years old | 242 (16.3) |
| University | 1184 (80.5) |
| Undisclosed | 7 (0.5) |

| Index of Multiple Deprivation (IMD) | |
|---|---|
| | n (%) |
| Most Deprived | 10 (2.3) |
| 2 | 20 (4.65 |
| 3 | 37 (8.4) |
| 4 | 43 (9.7) |
| 5 | 39 (8.8) |
| 6 | 62 (14.0) |
| 7 | 56 (12.6) |
| 8 | 54 (12.2) |
| 9 | 44 (9.9) |
| Least Deprived | 78 (17.6) |

| Participant report of household income | |
|---|---|
| | n (%) |
| Less than 5,000 | 9 (0.6) |
| 5,001 – 10,000 | 15 (1.0) |
| 10,001 – 20,000 | 39 (2.7) |
| 20,001 – 30,000 | 92 (6.3) |
| 30,001 – 50,000 | 296 (20.4) |
| 50,001 – 75,000 | 383 (26.4) |
| 75,001 – 100,000 | 275 (19.0) |
| 100,001 – 150,000 | 161 (11.1) |
| 150,001- 200,000 | 48 (3.3) |
| 200,001 – 250,000 | 7 (0.5) |
| Greater than 250,001 | 11 (0.8) |
| Undisclosed | 113 (7.8) |

Figure 3: Bubble map depicting spread of participations location in the UK (if postcode was provided) with respondent's education level, Index of Multiple Deprivation (IMD) and household income breakdown reported on the right.

149x144mm (330 x 330 DPI)

Suppl file 1: Full Questionnaire

# CoCoPIP supplementary information

**COVID-19 items** .......... **2**
*Situational influences* .......... *2*
*Health report* .......... *6*
*COVID Impact* .......... *13*
*COVID Concern* .......... *13*
*Social Distance Impacts* .......... *14*
*Vaccines* .......... *18*
**Parental Information** .......... **20**
*Caregiving support* .......... *20*
*Pregnancy related anxiety questionnaire – COVID adapted.* .......... *21*
*Parent infant attachment* .......... *22*
**Pregnancy information** .......... **23**
*Healthcare support and access* .......... *23*
**Parenting, family and home environment** .......... **27**
*Face-to-face interaction index.* .......... *27*
*Household space and environment* .......... *31*
*Family demographics and socioeconomic status* .......... *33*

For peer review only


For peer review only - http://bmjopen.bmj.com/site/about/guidelines.xhtml

## COVID-19 items

### Situational influences

We understand that guidance around COVID-19 has been changing, information is/was not always available and variability in health, work and family matters influence individual responses. We will ask you to share with us how local COVID-19 guidance impacted your lifestyle and behaviour.

Think back over the last few months: when you first changed your behaviour due to COVID-19 (start), during the time when there were the most rules and restrictions in your area related to containing COVID-19 (peak), and the gradual relaxing of those rules (reopening).

First, help us understand the timing of when COVID-19 **affected your life.** No need to look it up or be precise, just use your memory to tell us about **what date you pinpoint the following events (dd/mm/yyyy):**

a. Start of COVID-19
b. Peak of COVID-19
c. First day of Reopening

Currently according to the UK government guidance, what local restriction tier has your area been placed in?

a. Tier 1
b. Tier 2
c. Tier 3
d. Tier 4
e. Tier 5
f. I do not live in the UK
g. Not applicable

For peer review only

**How do the following statements reflect your response and behaviour with respect to changing local COVID-19 guidance?**

+Essential goods: food, hygiene, cleaning supplies and medicine

*Social distance: interact with others **further** than public health advice [1-2 m or 6 feet]

**Social contact: interact with others **closer** than public health advice

| Not true at all (1) | True at the Start of COVID-19 (2) | True at the peak of COVID-19 (3) | True during reopening (4) | True during the last month (5) |
|---|---|---|---|---|

a. Stayed home or on my property most of the time
b. Stayed home except for obtaining essentials goods+
c. Stayed home except for exercising and essential goods
d. Go on outings alone or with household members, but not seeking social contact with others
e. Social distance* contact with family or friends outside my immediate household
f. Social contact** with limited families or friends outside household
g. Not able to adhere to social distancing in public
h. Social contact** was necessary for my work

For peer review only

Think about when you first heard about COVID-19. What did your local government call the laws or ordinances guiding the changes in conduct, business closure and distance during social interactions?

a. Lockdown
b. Stay-at-home
c. Other

When did you modify your routine to adhere to lockdown

a. I have largely kept my routine the same as before
b. At least a week before the government formally issued it
c. A few days before the government issued it
d. The day the government issued it
e. A few days after the government issued it
f. At least a week after the government issued it

Many regions have lifted or relaxed some of these restrictions. When did you modify your routine to return to normal?

d. At least a week before the government eased restrictions
e. A few days before the government eased restrictions
f. The day the government eased restrictions
g. A few days after the government eased restrictions
h. At least a week after the government eased restrictions
i. I have largely kept my routine the same as before
j. Not applicable

For peer review only

How hard was the initial adjustment to a new routine for you, your partner and family during lockdown?

| No adjustment (1) | Not hard, Easy or minor (2) | Hard yet Manageable (3) | Quite Hard, Challenging (4) | Unbearable, Devastating (5) |
|---|---|---|---|---|

Working remotely

Being responsible for homeschooling

Interfacing with online education or technology for myself, work or my child

Adjusting to lack of or limited childcare

Enforcing new routines and schedules for the family

Missing interactions with loved ones, colleagues and friends in person

How would you describe your current living situation regarding COVID-19?

a. Staying home at all times
b. Staying home except for getting food / medication
c. Staying home except for exercising and getting food / medication
d. Going out of the house for work or for other reasons, but avoiding social contact with others
e. Enjoying social contact with others while adhering to social distancing
f. Not engaging in social distancing

For peer review only

## Health report

Have you been diagnosed with COVID-19? Select the statement best reflects your experience.

a. No symptoms or reason to suspect I had / have it
b. Asymptomatic, but positive antibody or COVID-19 test
c. May have been exposed from contact with someone who had it, but no symptoms developed
d. Mild, cold-like symptoms or loss of smell, did not much impact daily function
e. Mild to moderate, flu-like symptoms, impaired daily function
f. Moderate, requiring bedrest, impacted daily function
g. Severe, requiring hospital treatment, but not life threatening
h. Life-threatening/critical with no prospect of long-term impact/disability
i. Life-threatening/critical with potential long-term impact/disability

Have you been diagnosed with COVID-19? Select the statement best reflects your experience.

j. No symptoms or reason to suspect I had / have it
k. Asymptomatic, but positive antibody or COVID-19 test
l. May have been exposed from contact with someone who had it, but no symptoms developed
m. Mild, cold-like symptoms or loss of smell, did not much impact daily function
n. Mild to moderate, flu-like symptoms, impaired daily function
o. Moderate, requiring bedrest, impacted daily function
p. Severe, requiring hospital treatment, but not life threatening
q. Life-threatening/critical with no prospect of long-term impact/disability
r. Life-threatening/critical with potential long-term impact/disability



For peer review only - http://bmjopen.bmj.com/site/about/guidelines.xhtml

Suppl file 1: Full Questionnaire

Do you think someone in your household has had, possibly had or currently has COVID-19 (with or without having test results)?

a. Partner
b. Child
c. Other household member
d. No members of my household

How severe were/are their symptoms/disease? Please select only one.

a. No symptoms
b. Mild, cold-like symptoms, did not much impact daily function
c. Mild to moderate, flu-like symptoms, impaired daily function
d. Moderate, requiring bedrest, impacted daily function
e. Severe, requiring hospital treatment, but not life threatening
f. Life-threatening/critical with no prospect of long-term impact/disability
g. Life-threatening/critical with potential long-term impact/disability
h. They lost their life

For peer review only

Select the statement that best reflects the experience of your lockdown with COVID-19 symptoms.

a. No symptoms, but positive antibody or COVID-19 test
b. May have been exposed from contact with someone who had it, but no symptoms developed
c. Mild, cold-like symptoms or loss of smell, did not much impact daily function
d. Mild to moderate, flu-like symptoms, impaired daily function
e. Moderate, requiring bedrest, impacted daily function
f. Severe, requiring hospital treatment, but not life threatening
g. Life-threatening/critical with no prospect of long-term impact/disability
h. Life-threatening/critical with potential long-term impact/disability
i. They lost their life

Select the statement that best reflects the experience of your lockdown

a. No symptoms, but positive antibody or COVID-19 test
b. May have been exposed from contact with someone who had it, but no symptoms developed
c. Mild, cold-like symptoms or loss of smell, did not much impact daily function
d. Mild to moderate, flu-like symptoms, impaired daily function
e. Moderate, requiring bedrest, impacted daily function
f. Severe, requiring hospital treatment, but not life threatening
g. Life-threatening/critical with no prospect of long-term impact/disability
h. Life-threatening/critical with potential long-term impact/disability
i. They lost their life

For peer review only

Do you think any one else significant to you *outside your household* has had or currently has COVID-19 or related symptoms (with or without having test results)? Please specify who and their relation to you:

a. No one I am close with
b. Parent
c. Child
d. Close relative (specify)
e. Close friend or community member (specify)
f. Other loved one (specify)

How were they diagnosed or affected by COVID-19? Select the statement that best reflects their experience.

a. No symptoms, but positive antibody or COVID-19 test
b. May have been exposed from contact with someone who had it, but no symptoms developed
c. Mild, cold-like symptoms or loss of smell, did not much impact daily function
d. Mild to moderate, flu-like symptoms, impaired daily function
e. Moderate, requiring bedrest, impacted daily function
f. Severe, requiring hospital treatment, but not life threatening
g. Life-threatening/critical with no prospect of long-term impact/disability
h. Life-threatening/critical with potential long-term impact/disability
i. They lost their life
j. Other relevant information (key worker, in nursing home, health care provider, etc.)



For peer review only - http://bmjopen.bmj.com/site/about/guidelines.xhtml

How has this affected you emotionally? Please rate how hard you found it / are finding it to cope with them having COVID-19:

a. Unbearable, not coping well
b. Very hard, challenging and distressed
c. Hard, but maintaining perspective
d. Not hard, but not an easy adjustment
e. Not hard at all

How well supported do you feel by your spouse/partner during the COVID-19 lockdown?

a. Extremely
b. Very much
c. Somewhat
d. Not very
e. I do not feel supported at all

How well supported do you feel by your friends and family during the COVID-19 lockdown?

a. Extremely
b. Very much
c. Somewhat
d. Not very
e. I do not feel supported at all

For peer review only

How difficult have you found it to be separated from these loved ones because of COVID-19?

a. Unbearable
b. Very difficult
c. Moderately difficult
d. Slightly difficult
e. Not affecting you at all

The next series of questions will be related to whether you have had to self-isolate and your experience during your period of self-isolation. Have you self-isolated in response to potential COVID-19 exposure?

a. Yes
b. No

Did you self-isolate with a partner or other family members?

a. Yes (please specify)
b. No

How many days did you self-isolate for?
(numeric response)

Were you able to self-isolate in your own home?

a. Yes
b. No (please specify)

For peer review only

How supported did you feel during self-isolation? (please let us know why)

a. not supported at all

b. a little supported

c. somewhat supported

d. very supported

How did you manage caregiving during self-isolation? (e.g., caring for your family) (open ended)

Do you use the NHS track and trace app?

a. Yes

b. No

Approximately how many times have you used this app to 'check-in' to a venue?

| | Number of times use<br>0 10 20 30 40 50 60 70 80 90 100 |
|---|---|
| In the last week () | 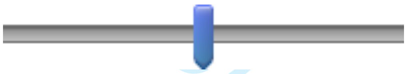 |
| In the last month () | 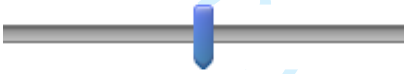 |

For peer review only

Suppl file 1: Full Questionnaire

## COVID Impact

Relative to others, rate how hard you find it to cope with the impact of COVID-19 on the following aspects of your life:

| Not hard at all, coping well (1) | Not hard, but not an easy adjustment (2) | Hard, but maintaining perspective (3) | Very hard, challenging & distressed (4) | Unbearable, not coping well (5) |
|---|---|---|---|---|

Physical health
Psychological well-being
Social life (or lack thereof)
Financial uncertainty
Disruption to routine
Adjustments to household
Changes in employment

## COVID Concern

When answering the next set of questions, please answer them in relation to your feelings since your government implementd a 'stay-at-home' or lockdown request because of COVID-19.

| Not at all (1) | A little (2) | Somewhat (3) | Quite a bit (4) | Very much (5) | All the time (6) |
|---|---|---|---|---|---|

I think about the effect of COVID-19 on my own physical health.
I worry about spreading COVID-19 to others.
I am concerned about the effects of lockdown on society.
Lockdown affects/affected my mental health.
I am afraid of COVID-19.
I am not worried about COVID-19.
I am worried that I or people I love will get sick from COVID-19.
I am stressed around other people because I worry I'll catch COVID-19.
I have tried hard to avoid other people because I don't want to get sick
I have tried hard to avoid other people because I don't want to make others sick.


For peer review only - http://bmjopen.bmj.com/site/about/guidelines.xhtml

## Social Distance Impacts

How much do each of the following statements reflect your attitude or experience during COVID-19?

| Not at all (1) | A little (2) | Somewhat (3) | Quite a bit (4) | Very true (5) |
|---|---|---|---|---|

I spend more time alone

There is a sense of solidarity in my community

I am even busier than before.

I have volunteered to get groceries for neighbors or acquaintances

I feel grateful for my health

I have more time than usual

There is more fighting among the members of my household

I have picked up a new hobby

I am learning a new skill

I appreciate moments of reprieve from the chaos in my home and outside it.

Technology brings us together during these turbulent

I spend my leisure time more intentionally times.

I am exercising or appreciating outdoor time more often.

I feel proud of my community's response

I am hopeful that we can learn something out of this.

I am disappointed by the changes in observing special occasions and holidays

I am concerned for the health of an elderly or vulnerable family or friend due to COVID-19. I am concerned for the well-being of a family or friend due to social distancing.

I am now more conscious about my personal space boundaries when outside my home.

I am just as cautious and vigilant in maintaining distance from others in public as I was at the start of the outbreak

For peer review only

Do you think someone in your household has had, possibly had or currently has COVID-19 (with or without having test results)?

e. Partner

f. Child

g. Other household member

h. No members of my household

How severe were/are their symptoms/disease? Please select only one.

i. No symptoms

j. Mild, cold-like symptoms, did not much impact daily function

k. Mild to moderate, flu-like symptoms, impaired daily function

l. Moderate, requiring bedrest, impacted daily function

m. Severe, requiring hospital treatment, but not life threatening

n. Life-threatening/critical with no prospect of long-term impact/disability

o. Life-threatening/critical with potential long-term impact/disability

p. They lost their life

For peer review only

Select the statement that best reflects the experience of your lockdown with COVID-19 symptoms.

j. No symptoms, but positive antibody or COVID-19 test
k. May have been exposed from contact with someone who had it, but no symptoms developed
l. Mild, cold-like symptoms or loss of smell, did not much impact daily function
m. Mild to moderate, flu-like symptoms, impaired daily function
n. Moderate, requiring bedrest, impacted daily function
o. Severe, requiring hospital treatment, but not life threatening
p. Life-threatening/critical with no prospect of long-term impact/disability
q. Life-threatening/critical with potential long-term impact/disability
r. They lost their life

Select the statement that best reflects the experience of your lockdown

j. No symptoms, but positive antibody or COVID-19 test
k. May have been exposed from contact with someone who had it, but no symptoms developed
l. Mild, cold-like symptoms or loss of smell, did not much impact daily function
m. Mild to moderate, flu-like symptoms, impaired daily function
n. Moderate, requiring bedrest, impacted daily function
o. Severe, requiring hospital treatment, but not life threatening
p. Life-threatening/critical with no prospect of long-term impact/disability
q. Life-threatening/critical with potential long-term impact/disability
r. They lost their life

Do you think any one else significant to you *outside your household* has had or currently has COVID-19 or related symptoms (with or without having test results)? Please specify who and their relation to you:



For peer review only - http://bmjopen.bmj.com/site/about/guidelines.xhtml

g. No one I am close with

h. Parent

i. Child

j. Close relative (specify)

k. Close friend or community member (specify)

l. Other loved one (specify)

How were they diagnosed or affected by COVID-19? Select the statement that best reflects their experience.

k. No symptoms, but positive antibody or COVID-19 test

l. May have been exposed from contact with someone who had it, but no symptoms developed

m. Mild, cold-like symptoms or loss of smell, did not much impact daily function

n. Mild to moderate, flu-like symptoms, impaired daily function

o. Moderate, requiring bedrest, impacted daily function

p. Severe, requiring hospital treatment, but not life threatening

q. Life-threatening/critical with no prospect of long-term impact/disability

r. Life-threatening/critical with potential long-term impact/disability

s. They lost their life

t. Other relevant information (key worker, in nursing home, health care provider, etc.)

For peer review only



For peer review only - http://bmjopen.bmj.com/site/about/guidelines.xhtml

How has this affected you emotionally? Please rate how hard you found it / are finding it to cope with them having COVID-19:

f. Unbearable, not coping well
g. Very hard, challenging and distressed
h. Hard, but maintaining perspective
i. Not hard, but not an easy adjustment
j. Not hard at all

## Vaccines

Are you currently eligible for the COVID-19 vaccine?

a. Yes
b. No
c. Unsure
d. Prefer not to answer
e. Not applicable

Have you and/or your partner been vaccinated?

a. Yes
b. No
c. Not applicable

Have any of your family members or loved ones been vaccinated?

a. Yes (please specify who)
b. No
c. Unsure

For peer review only


For peer review only - http://bmjopen.bmj.com/site/about/guidelines.xhtml

Suppl file 1: Full Questionnaire

Do you think that getting vaccinated has changed the way you socialise?

a. Yes
b. No
c. Prefer not to answer

Could you tell us how and/or why getting vaccinated has changed the way you socialise? (open ended)

For peer review only

## Parental Information

### Caregiving support

Please tell us who has served as caregivers to your baby since their birth. Please select all that apply.

| Every day (1) | A few times a week (2) | Several times a month (3) | Once a month (4) | Never or less than once a month (5) |
|---|---|---|---|---|

a. My partner/spouse
b. My parents or in-laws
c. My grandparents or grandparents-in-law
d. Other relatives (siblings, aunts/uncles, cousins etc.)
e. Close friends
f. Nanny/Babysitter
g. Other (please specify)

During an average night, how many hours does your baby sleep? (scale 0-8 hours)

a. Before COVID-19
b. Since COVID-19

If your baby sleeps more than 8 hours, please specify below:
(open ended)

On an average day during the week, how many hours would you say you spend with your baby between 7am and 7pm? (scale 0-12 hours)

a. Before COVID-19
b. Since COVID-19

In an average month, how many people other than caregivers and household members have interacted with your baby? (scale 0-30)

a. Before COVID-19 (number of people
b. Since COVID-19 (number of people)



For peer review only - http://bmjopen.bmj.com/site/about/guidelines.xhtml

When you and your baby are together, how often do you:

| A lot (1) | Often (2) | Sometimes (3) | Rarely (4) | Never (5) |
|---|---|---|---|---|

a. Hold your baby
b. Sing to your baby
c. Soothe your baby
d. Talk to your baby
e. Read to your baby
f. Imitate your baby
g. Bathe your baby
h. Stroke your baby
i. Cuddle your baby
j. Kiss your baby
k. Play with your baby (with toys)
l. Play face to face with your baby (without toys)
m. Play with your baby (with screens, e.g. iPad)
n. Play with your baby (using household objects)
o. Watch your baby play
p. Watch your baby sleep

a. Take your baby outside
b. Take your baby for a walk in a pram/stroller

## Pregnancy related anxiety questionnaire – COVID adapted.

We are going to ask how much you think or worry about **your pregnancy or the experience of your pregnant partner during birth.** Please slide the bar that most closely matches your feelings about each statement.

| Not at all (1) | A little (2) | Somewhat (3) | Quite a bit (4) | Very much (5) |
|---|---|---|---|---|

a. I worry about the pain of contractions
b. I worry about the pain of delivery
c. I worry about not being in control of my body while I give birth, or how my partner will respond while giving birth (shouting, crying, bowel movement, etc.)
d. I think about coming in contact with COVID-19


For peer review only - http://bmjopen.bmj.com/site/about/guidelines.xhtml

e. I think about the pain from getting COVID-19
f. I think about difficulty accessing health care if there is a virus spreading
g. I think that something will be wrong with my baby physically
h. I think that something will be wrong with my baby mentally
i. I think I will transmit stress about COVID-19 to my baby
j. I think due to COVID-19 my baby may be held back
k. I think due to COVID-19 my baby may not be normal
l. I think due to COVID-19 my baby may not get proper medical attention
m. I think due to COVID-19 my birth will have complications
n. I think that after birth my child will be in poor health
o. I think about creating a virus-free home for my child
p. I think about creating a safe, healthy home for my child
q. Thinking about COVID-19 makes me feel threatened
r. I think my baby will not be healthy due to COVID-19
s. I think I can transmit COVID-19 to my baby
t. COVID-19 has made me rethink my family planning

### Home schooling

'Could you describe your experience of home schooling during your pregnancy or as a new parent?'

(open ended)

### Parent infant attachment

Could you tell us a little about what you imagine your baby will be like and how the two of you will get along?

(open ended)

Could you tell us a little about what your baby and how the two of you get along?

(open ended)



For peer review only - http://bmjopen.bmj.com/site/about/guidelines.xhtml

Suppl file 1: Full Questionnaire

## Pregnancy information

### Healthcare support and access

Have you attended some or all midwife, doctor or OB-GYN pregnancy appointments in person?

a. Yes

b. No

Do you feel comfortable attending your pregnancy appointments?

a. Yes
b. A little
c. Unsure
d. Not at all

Could you please tell us why you chose the answer you did for the last question? (open ended)

Have you been offered online, phone or video call midwife appointments?

a. Yes

b. No

Do you feel that talking to your midwife online has allowed you to ask the questions you've wanted to and made you feel at ease?

a. Yes
b. A little
c. Unsure
d. Not at all

Could you tell us why you chose the answer you did for the last question? (open ended)


For peer review only - http://bmjopen.bmj.com/site/about/guidelines.xhtml

How well supported do you feel by your midwife, doctor (OB-GYN) and other prenatal healthcare professionals during this time?

a. Extremely

b. Very much

c. somewhat

d. not very

e. I do not feel supported at all

Could you tell us about the support from your healthcare providers during your pregnancy? (open ended)

When attending your pregnancy appointments, have you worn a mask, gown or other form of personal protective equipment (PPE)? Check all that apply.

a. No, I nor my healthcare providers used PPE

b. No I did not, though the healthcare staff did wear PPE

c. The healthcare staff provided me with PPE (please specify, gloves, gown, etc.)

d. I brought a face covering or mask with me to the appointment

e. I brought additional PPE with me to the appointment (please specify, gloves, gown, etc.)

How comfortable did you feel interacting with your healthcare providers at these appointments?
(open ended)

For peer review only

Have you had any appointments cancelled or rescheduled?

a. No
b. Rescheduled by health provider
c. Cancelled by health provider
d. I did not attend due to safety or health concerns
e. I cancelled due to other reasons
f. I have not booked any appointments since COVID-19 due to safety or health concerns

Are you attending antenatal classes? (Either online or in person)

a. Yes (in person)
b. Yes (online)
c. No

Are the classes you are taking offered by public, private and alternative healthcare providers? Please tick and specify what kind of classes you took.

a. Private antenatal class (e.g. NCT classes, small business, offered by university or employer, etc.)
b. Not private, provided by or subsidized by the public healthcare system (e.g. NHS, Medicare)
c. Mental health services (counselling, family planning, genetic counselling, etc.)
d. Other, including alternative wellness services (yoga, doula, etc.)

For peer review only

In your own words, please tell us about these classes; how often you attend and how helpful you have found them.

(open ended)

Are these classes supporting you in the same way (if you had a previous pregnancy) or in the way you expected during your pregnancy?

a. Yes
b. No (please specify)
c. I am not sure

For peer review only



For peer review only - http://bmjopen.bmj.com/site/about/guidelines.xhtml

## Parenting, family and home environment

### Face-to-face interaction index.

Think about who your baby has interacted with so far, and who would have interacted with your baby in person if not for COVID-19. We want to get an understanding of your baby's visual and physical social interactions. Who would you have expected to see your baby (visit, talk, make silly faces, play from afar) or hold your baby (e.g. rock, play with your baby)? Please select all that apply.

| Actually interacted with my baby (1) | | | If not for COVID-19, would have interacted with my baby (2) | | |
|---|---|---|---|---|---|
| See from afar (1.1) | Hold up close (1.2) | Via videochat (1.3) | See from afar (2.1) | Hold up close (2.2) | Via videochat (2.3) |

a. My partner
b. My parents
c. My in-laws
d. My or my partner's grandparents
e. Other adult relatives
f. Friends
g. Colleagues
h. Nanny/Babysitter
i. Neighbors
j. Healthcare or prenatal service provider
k. Other children

Think about how often you and your baby have interacted with friends and family, both in person and on video in 2020. Think about the time **before COVID-19, right after the start of COVID-19 in your region, and in the last month.**



For peer review only - http://bmjopen.bmj.com/site/about/guidelines.xhtml

Suppl file 1: Full Questionnaire

How often did you (without your baby) videochat with others (for work, family or social reasons)?

| Never (1) | A handful of times (2) | At least once a month (3) | At least once a week (4) | At least once day (5) | Multiple times a day (6) | Not Applicable (7) |
|---|---|---|---|---|---|---|

a. Before COVID-19
b. Start of COVID-19
c. Last month

In a typical month, how often did you record videos of your baby using your smartphone?

| Never (1) | A handful of times (2) | At least once a month (3) | At least once a week (4) | At least once day (5) | Multiple times a day (6) | Not Applicable (7) |
|---|---|---|---|---|---|---|

a. Before COVID-19
b. Start of COVID-19
c. Last month

How often did you AND your baby interact in person with friends or family, other than caregivers?

| Never (1) | A handful of times (2) | At least once a month (3) | At least once a week (4) | At least once day (5) | Multiple times a day (6) | Not Applicable (7) |
|---|---|---|---|---|---|---|

a. Before COVID-19
b. Start of COVID-19
c. Last month


For peer review only - http://bmjopen.bmj.com/site/about/guidelines.xhtml
For peer review only

Using your phone, do you share videos or pictures of your baby with (select all that apply):

a. your sibling(s)
b. your parents (baby's grandparents)
c. other relatives
d. friends
e. colleagues
f. private online network (e.g., baby website)
g. public online network (e.g., personal blog)
h. private social media (e.g., instagram, facebook)
i. public social media (e.g., instagram, facebook)
j. None of the above

How responsive is your baby to others during interactions on videochat, Zoom, facetime or webcam?

a. Very responsive
b. Quite responsive
c. Somewhat
d. Rarely
e. Never responds
f. Not applicable

For peer review only

Suppl file 1: Full Questionnaire

How responsive is your baby to videos, music or other media played from your mobile device?

a. Most of the time
b. Quite a bit
c. Somewhat
d. Rarely
e. Never responds
f. Not applicable

Have you captured videos of your baby's milestones or behaviours? Please select all that apply

a. No
b. Reaching
c. Grasping
d. Sitting up
e. Rolling over
f. Smiling
g. Babbling
h. Waving
i. Other

For peer review only


For peer review only - http://bmjopen.bmj.com/site/about/guidelines.xhtml

Suppl file 1: Full Questionnaire

## Household space and environment

Have you been living and/or staying in the same home since Feb 2020? Since this time have you:

a. Moved home
b. Visited friends or family
c. Gone to stay with family
d. Gone on holiday
e. Other (please specify)
f. None of the above

How many people lived with you in your current residence?

| Before Covid-19 (1) | Peak Covid-19 (2) | Presently (3) |
|---|---|---|

Number of Adults

Number of Children (under the age of 18)

Number of adults with conditions which confer risk for or complications with COVID-19

How many rooms within your current residence do you feel comfortable relaxing or spending time in that are not your bedroom? This can also include outdoor spaces that are on your property.

a. < 2
b. 2-3
c. 3-5
d. 5-8
e. 8+


For peer review only - http://bmjopen.bmj.com/site/about/guidelines.xhtml

Suppl file 1: Full Questionnaire

Do you currently have access to outside space you feel safe spending time in? Please select all that apply.

a. Balcony or backyard
b. Communal garden or yard
c. Small private garden or yard
d. Large private garden or yard
e. Allotment
f. Parks
g. Playgrounds
h. Nearby countryside or field
i. National park, beach or other outdoor space
j. Other (please specify)

In your home, how many televisions are there in total?

a. < 2
b. 2-3
c. 3-5
d. 5-8
e. 8+

In your home, how many mobile phones are presently in use?

a. < 2
b. 2-3
c. 3-5
d. 5-8
e. 8+

For peer review only

Suppl file 1: Full Questionnaire

How many personal computers and/or tablets are presently in use?

a. < 2
b. 2-3
c. 3-5
d. 5-8
e. 8+

## Family demographics and socioeconomic status

Are you or your partner currently pregnant?

a. Yes
b. No

Have you had your first trimester scan?

a. Yes
b. No

Are you the parents to an infant under the age of 7 months?

a. Yes
b. No

How old is your child?

a. Months
b. Days

Who is completing this questionnaire?

a. Mother
b. Father
c. Non birth mother
d. Other (please specify)

For peer review only

What is your ethnic background? You may select more than one.

a. White
b. Black
c. Asian
d. Mixed/multiple ethnic group (e.g. white and African)
e. Hispanic
f. Arab
g. Other (please specify)
h. Prefer not to answer

How old are you?

(numeric response)

What is the mother's highest level of education?

a. High School/ Secondary School (or something equivalent) to age 16
b. College level education (to age 18)
c. Undergraduate degree
d. Postgraduate degree (Masters, PhD)

What is the father's/partner's highest level of education?

a. High School/ Secondary School (or something equivalent) to age 16
b. College level education (to age 18)
c. Undergraduate degree
d. Postgraduate degree (Masters, PhD)

What is your post code?

(open ended optional)



For peer review only - http://bmjopen.bmj.com/site/about/guidelines.xhtml

What is your current employment status? Please select ALL that apply

a. On maternity leave
b. Unemployed (or job seeking)
c. Unemployed due to COVID-19
d. Full-time home-maker
e. Retired
f. Employed full-time (working from home due to COVID-19)
g. Employed full-time (I am a key worker)
h. Employed full time (with reduced hours and salary loss)
i. Employed full-time (with reduced hours but without salary loss)
j. Employed with reduced hours due to childcare/homeschooling needs as a result of COVID-19
k. Employed (part-time) with reduced hours and salary loss
l. Employed (part-time) with reduced hours but without salary loss
m. Furloughed (with salary) - Full-time
n. Furloughed (without salary) - Full-time
o. Furloughed (with salary) - Part-time
p. Furloughed (without salary) - Part-time
q. Self-employed (currently still working)
r. Self-employed (unable to work due to COVID-19)
s. Self-employed with reduced hours and salary loss
t. Self-employed with reduced hours but without salary loss
u. Student - My studies have not been affected by the COVID-19
v. Student - I have had to postpone my studies due to COVID-19
w. Student - My studies have been negatively impacted by the COVID-19
x. Other (please specify)



For peer review only - http://bmjopen.bmj.com/site/about/guidelines.xhtml

| Measure (items) | Description | Scale | Example item |
|---|---|---|---|
| **COVID-19 items** | | | |
| Situational influences | Assessing adjustment and adherence to government guidelines. | | 'When did you modify your routine to adhere to the social distancing guidance?' |
| Health report | Assessing risk for COVID exposure and symptoms of parents, partner and/or social network. As well as self-isolation behaviours and support (if applicable) | | 'Have you been diagnosed with COVID?' 'How supported did you feel during self-isolation?' |
| COVID Impact (7 items) | How COVID-19 has impacted themselves and their household (e.g., physical health, disruption to routine). | Five-point scale from 'not hard at all, copying well' to 'unbearable, not coping well' | |
| COVID Concern (10 items) | | Six-point scale from 'not at all' to 'all the time' | 'I am not worried about COVID-19' |
| Event Impact Scale-Revised (20 items) † | Assessing the distress experience as a result of traumatic events | Five-point scale from 'extremely' to 'not at all' | 'I felt irritable and angry' |
| Social distance impacts | Assessing participants attitudes and experience during COVID-19. | Five-point scale from 'not at all' to 'very true' | 'I spend my leisure time more intentionally' |
| Vaccines | Assessing access to and attitude towards a vaccine and potential changes in social behaviour in relation to a vaccine. | | 'Are you currently eligible for the COVID-19 vaccine?' / 'Could you tell us how and/or why getting vaccinated has changed the way you socialise?' |
| **Parental information** | | | |
| State-Trait Anxiety state (STAI-S) (20 items) † | Assessing the presence and severity of an individual's state-based anxiety (i.e., anxiety in response to what is happening right now). | Four-point scale from 'not at all' to 'very much so' | 'I feel strained' |
| Caregiving support | Questions assessing remote and in person availability of household members, partners, and social network of families | | |
| Home schooling | Exploring the additional demands on parents in the home and support available for home schooling. | | 'Could you describe your experience of home schooling during your pregnancy or as a new parent?' |
| Stress Life Inventory (43 items) † | Assessing the number of stressful life events experienced by the family over a 12-month period | Items rates 'yes' or 'no' | 'Being fired at work' |

For peer review only

| | | | |
|---|---|---|---|
| Pregnancy related anxiety questionnaire (PRAQ) (20 items)* | Assessing pregnancy-specific anxiety in women during the antenatal period. | Five-point scale from 'not at all' to 'very much' | 'I think that after birth my child will be in poor health' / 'I think about creating a virus-free home for my child' |
| **Pregnancy information** | | | |
| Healthcare support and access | Exploring parental experiences of their healthcare support and care, and access to antenatal routine care during the pandemic | | 'Do you feel comfortable attending your pregnancy appointments?'/ 'Could you tell us about the support from your healthcare provides during your pregnancy' |
| Antenatal Emotional Attachment Scale (AEAS) - Maternal (19 items) † | Questions asking expectant parents about their thoughts and feelings over the last two-week period about their baby | Responses on a varying five-point scale (e.g., 'almost all the time' to 'not at all' and 'very positive' to 'very negative') | 'I think of the developing baby as mostly as;' |
| Antenatal Emotional Attachment Scale (AEAS) – Paternal (16 items) † | | | 'I have found myself touching, or rubbing with my hand, the outside of my partners stomach where the baby is:' |
| **Toddler outcomes** | | | |
| Infant Behaviour Questionnaire (IBQ) – short form (36 items) † | Measures the dimensions of temperament in children younger than 12 months of age | Eight-point scale from 'never' to 'always' (including option for 'does not apply') | 'When tired, how often did your baby show distress?' |
| Infant/Toddler Sensory Profile (ITSP) (36 items) † | A caregiver report on how responsive, sensitive, avoidant and alert their babies are when presented with stimuli visual, auditory, tactile and vestibular modalities | Six-point scale from 'almost always' to 'almost never' (including an option for 'does not apply') | 'My child is active throughout the day' |
| Ages and Stages (ASQ-3) (39 items) † | A developmental screening tool assessing a child's developmental progress in six domains (i.e., communication, gross motor, fine motor, problem solving and personal-social development). | Three-point scale from 'yes' to 'not yet' | 'When your baby wants something, does he tell you by pointing to it?' |
| Oxford Communication Development Inventory – 100 words (CDI) † | A UK-adapted measure of toddler language development | Two-point scale; 'understands' and 'understands and says' | 'car' |
| Quantitative Checklist for Autism in Toddlers (Q-CHAT) (25 items) † | A quantitative measure of early autistic traits in toddlers | Responses given on a varying five-point scale (e.g., 'many times a day to never' and 'always' to 'never') | 'How easy is it for your child to adapt when his/her routine changes or when things are out of their usual place?' |
| Vineland parent and caregiver form† | A number of questions about the home environment and family-life behaviour | Three-point scale from "usually or often' to 'never' | 'When you look or point at something, looks in that direction.' |

| Parenting, family and home environment | | | |
|---|---|---|---|
| Household space and environment | Assessing access to outdoor/indoor space and technology | | 'Do you currently have access to outdoor space you feel safe spending time in?' / 'In your home, how many mobile phones are presently in use?' |
| Parent-infant attachment | Parental description of relationship with infant (imagined and actual) | | 'Could you tell us a little about what you image your baby will be like and how the two of you will get along?' |
| Face-to-face interaction index | Exploring the frequency of face-to-face and distant interaction the infant has had since birth | | 'How often did you with/without your baby video chat with others?'/ 'How responsive is your baby to others during virtual interactions?' |
| Parenting reflective functioning questionnaire (PRFQ) (18 item) † | A short measure assessing parental reflective functioning or mentalising. | Responses are rated on a seven-point scale from 'strongly disagree' to 'strongly agree' | 'I always know why my child acts the way he or she does' |
| Comprehensive early childhood parenting questionnaire (CECPAQ) (54 items) † | A parent report measure assessing five domains of parenting (i.e., support, stimulation, structure, harsh discipline and positive measure). | Responses are rated on a six-point scale from 'never' to 'always' | 'When my child is having a hard time, I am able to help him/her. |
| Difficulties in Emotion Regulation Scale (DERS) (19 items) † | A self-report measure of subjective emotion ability | Responses are rated on a five-point scale from 'almost never' to 'almost always' | 'When I'm upset, I can still get things done' |
| Family demographics and socioeconomic questions | Parent and infant age or gestation of pregnancy; sex; ethnicity; location; parent and partner highest level of qualification; employment status and household income. | | 'What is your highest level of education?' |
| Postcode | Used to derived indices of multiple deprivation. | | |

*several questions have been adapted for use during COVID-19

†These items have not been included within the supplementary information as they are standardised questionnaires and not specifically created for this study.

*Supp Table 1: A detailed summary of measures used within the study.*

For peer review only

For peer review only

**STROBE 2007 (v4) Statement—Checklist of items that should be included in reports of *cohort studies***

| Section/Topic | Item # | Recommendation | Reported on page # |
|---|---|---|---|
| **Title and abstract** | 1 | (*a*) Indicate the study's design with a commonly used term in the title or the abstract | 1-2 |
| | | (*b*) Provide in the abstract an informative and balanced summary of what was done and what was found | 2 |
| **Introduction** | | | |
| Background/rationale | 2 | Explain the scientific background and rationale for the investigation being reported | 4-7 |
| Objectives | 3 | State specific objectives, including any prespecified hypotheses | 7-8 |
| **Methods** | | | |
| Study design | 4 | Present key elements of study design early in the paper | 7-8 |
| Setting | 5 | Describe the setting, locations, and relevant dates, including periods of recruitment, exposure, follow-up, and data collection | 8-10 |
| Participants | 6 | (*a*) Give the eligibility criteria, and the sources and methods of selection of participants. Describe methods of follow-up | 8-10 |
| | | (*b*) For matched studies, give matching criteria and number of exposed and unexposed | n/a |
| Variables | 7 | Clearly define all outcomes, exposures, predictors, potential confounders, and effect modifiers. Give diagnostic criteria, if applicable | 11-16 |
| Data sources/ measurement | 8* | For each variable of interest, give sources of data and details of methods of assessment (measurement). Describe comparability of assessment methods if there is more than one group | 16 |
| Bias | 9 | Describe any efforts to address potential sources of bias | 8 |
| Study size | 10 | Explain how the study size was arrived at | 9 |
| Quantitative variables | 11 | Explain how quantitative variables were handled in the analyses. If applicable, describe which groupings were chosen and why | 15-16 |
| Statistical methods | 12 | (*a*) Describe all statistical methods, including those used to control for confounding | 15-16 |
| | | (*b*) Describe any methods used to examine subgroups and interactions | n/a |
| | | (*c*) Explain how missing data were addressed | n/a |
| | | (*d*) If applicable, explain how loss to follow-up was addressed | n/a |
| | | (*e*) Describe any sensitivity analyses | n/a |
| **Results** | | | |

For peer review only

| | | | |
|---|---|---|---|
| Participants | 13* | (a) Report numbers of individuals at each stage of study—eg numbers potentially eligible, examined for eligibility, confirmed eligible, included in the study, completing follow-up, and analysed | 16 |
| | | (b) Give reasons for non-participation at each stage | n/a |
| | | (c) Consider use of a flow diagram | 10 |
| Descriptive data | 14* | (a) Give characteristics of study participants (eg demographic, clinical, social) and information on exposures and potential confounders | 16 |
| | | (b) Indicate number of participants with missing data for each variable of interest | n/a |
| | | (c) Summarise follow-up time (eg, average and total amount) | 10 |
| Outcome data | 15* | Report numbers of outcome events or summary measures over time | 11 |
| Main results | 16 | (*a*) Give unadjusted estimates and, if applicable, confounder-adjusted estimates and their precision (eg, 95% confidence interval). Make clear which confounders were adjusted for and why they were included | n/a |
| | | (*b*) Report category boundaries when continuous variables were categorized | n/a |
| | | (*c*) If relevant, consider translating estimates of relative risk into absolute risk for a meaningful time period | n/a |
| Other analyses | 17 | Report other analyses done—eg analyses of subgroups and interactions, and sensitivity analyses | n/a |
| **Discussion** | | | |
| Key results | 18 | Summarise key results with reference to study objectives | n/a |
| **Limitations** | | | |
| Interpretation | 20 | Give a cautious overall interpretation of results considering objectives, limitations, multiplicity of analyses, results from similar studies, and other relevant evidence | n/a |
| Generalisability | 21 | Discuss the generalisability (external validity) of the study results | n/a |
| **Other information** | | | |
| Funding | 22 | Give the source of funding and the role of the funders for the present study and, if applicable, for the original study on which the present article is based | 23 |

*Give information separately for cases and controls in case-control studies and, if applicable, for exposed and unexposed groups in cohort and cross-sectional studies.

**Note:** An Explanation and Elaboration article discusses each checklist item and gives methodological background and published examples of transparent reporting. The STROBE checklist is best used in conjunction with this article (freely available on the Web sites of PLoS Medicine at http://www.plosmedicine.org/, Annals of Internal Medicine at http://www.annals.org/, and Epidemiology at http://www.epidem.com/). Information on the STROBE Initiative is available at www.strobe-statement.org.

For peer review only
